# Integrated bioinformatics and experiment revealed that cuproptosis is the potential common pathogenesis of three kinds of primary cardiomyopathy

**DOI:** 10.18632/aging.205298

**Published:** 2023-12-11

**Authors:** Mengxi Wang, Xiaozhuo Xu, Jianghong Li, Ziwei Gao, Yuhan Ding, Xiaohu Chen, Qian Xiang, Le Shen

**Affiliations:** 1 Department of Cardiology, Affiliated Hospital of Nanjing University of Chinese Medicine, Nanjing 210029, China; 2 Department of Cardiology, Jiangsu Province Hospital of Chinese Medicine, Nanjing 210029, China; 3 First Clinical Medical College, Nanjing University of Chinese Medicine, Nanjing 210023, China

**Keywords:** cuproptosis, primary cardiomyopathy, pathogenesis, diagnosis, molecular subtypes

## Abstract

Cuproptosis is a recently reported new mode of programmed cell death which might be a potential co-pathogenesis of three kinds of primary cardiomyopathy. However, no investigation has reported a clear relevance between primary cardiomyopathy and cuproptosis. In this study, the differential cuproptosis-related genes (CRGs) shared by three kinds of primary cardiomyopathy were identified in training sets. As a result, four CRGs shared by three kinds of primary cardiomyopathy were acquired and they were mainly related to biological processes such as cell death and immuno-inflammatory response through differential analysis, correlation analysis, GSEA, GSVA and immune cell infiltration analysis. Then, three key CRGs (K-CRGs) with high diagnostic value were identified by LASSO regression. The results of nomogram, machine learning, ROC analysis, calibration curve and decision curve indicated that the K-CRGs exhibited outstanding performance in the diagnosis of three kinds of primary cardiomyopathy. After that, in each disease, two molecular subtypes clusters were distinguished. There were many differences between different clusters in the biological processes associated with cell death and immunoinflammation and K-CRGs had excellent molecular subtype identification efficacy. Eventually, results from validation datasets and *in vitro* experiments verified the role of K-CRGs in diagnosis of primary cardiomyopathy, identification of primary cardiomyopathic molecular subtypes and pathogenesis of cuproptosis. In conclusion, this study found that cuproptosis might be the potential common pathogenesis of three kinds of primary cardiomyopathy and K-CRGs might be promising biomarkers for the diagnosis and molecular subtypes identification of primary cardiomyopathy.

## INTRODUCTION

Primary cardiomyopathy, characterized by pathological changes of cardiomyocytes and cardiac structure, is a disease with unknown cause after excluding various secondary factors [1]. Primary cardiomyopathy, including dilated cardiomyopathy (DCM), hypertrophic cardiomyopathy (HCM) and arrhythmogenic right ventricular cardiomyopathy (ARVC), could lead to heart failure and arrhythmia, and is an important cause of sudden death and heart transplantation [2]. In the past few years, more and more studies have focused on the pathogenesis of primary cardiomyopathy, which has gradually deepened the understanding of the pathogenesis of primary cardiomyopathy. Researchers have begun to realize that there may be common pathogenesis among various primary cardiomyopathy, but the specific molecular mechanism has not been fully elucidated [3–5]. In terms of diagnosis, the diagnosis of primary cardiomyopathy mainly relies on echocardiography and cardiovascular magnetic resonance [6, 7]. Usually, the patients diagnosed with cardiomyopathy by echocardiography or cardiovascular magnetic resonance have entered the middle or late stages of disease. At that time, the treatment measures mainly focused on improving the symptoms of heart failure and preventing malignant arrhythmia, which is difficult to achieve satisfactory efficacy [8, 9]. Early genetic diagnostic markers and targeted therapy methods need to be further developed. Thus, it can be seen, there are many difficulties in the pathogenesis, diagnosis and treatment of primary cardiomyopathy, and it is necessary to carry out related research.

Previous studies have shown that programmed cell death, including apoptosis, pyroptosis and ferroptosis, is associated with the occurrence and development of primary cardiomyopathy [10]. Recently, researchers have identified a new pattern of programmed cell death, that is, cuproptosis. The occurrence of cuproptosis depends on mitochondrial metabolism and cellular respiration, and its pathological features are the aggregation of lipoylated proteins and lack of iron-sulfur cluster proteins [11]. Some studies have shown that mitochondrial dysfunction is closely related to the pathogenesis of various cardiomyopathies [12, 13]. More importantly, significant copper transport dysfunction in cardiomyocytes has been observed in animal models of cardiomyopathy, and copper chelator could remedy this phenomenon and ameliorate cardiomyopathy-induced cardiac function decline [14]. In addition, other studies have found that serum copper levels in patients with cardiomyopathy are significantly increased, but the specific mechanism is not fully understood [15]. Hence, on the basis of the findings of previous research, we hypothesized that cuproptosis might be involved in the pathogenesis of multiple primary cardiomyopathies. Further studies are expected to elucidate the common pathogenesis of multiple primary cardiomyopathies, and may help to find diagnostic markers and therapeutic targets.

Bioinformatics analysis has the advantage of multi-dimension and multi-layer, which is of great value in investigating the pathogenesis, diagnosis and treatment of diseases. However, there was no research that employed this analysis technique to primary cardiomyopathy and cuproptosis. Therefore, through bioinformatics analysis and *in vitro* experiment, our investigation tried to explore the role of cuproptosis in the three kinds of primary cardiomyopathy, and determine the value of CRGs in the three kinds of primary cardiomyopathy for diagnosis and molecular subtype identification, for purpose of providing a new way to solve the dilemma of primary cardiomyopathy. The research procedure of our investigation was shown in the Supplementary Figure 1.

## MATERIALS AND METHODS

### Sources of data

The keywords “dilated cardiomyopathy”, “hypertrophic cardiomyopathy” and “arrhythmogenic right ventricular cardiomyopathy” were employed to seek the datasets related to primary cardiomyopathy in the Gene Expression Omnibus (GEO) database. After that, we screened the search results based on the following criteria: (1) The study samples were human ventricular tissue. (2) The subjects were patients with primary cardiomyopathy or healthy people. Finally, we selected the two datasets with the largest sample sizes for each primary cardiomyopathy, and a total of six datasets were included in this study. The GSE141910 and GSE29819 datasets were appointed as training sets, including 166 people with DCM, 28 people with HCM, six people with ARVC and 172 healthy people. The GSE57338, GSE36961, GSE107475 and GSE107156 datasets were appointed as validation sets, including 82 people with DCM, 106 people with HCM, nine people with ARVC and 180 healthy people. The detailed information of the six datasets was presented in the Supplementary Table 1. In addition, the CRGs included in this research were derived from previously published research [16–19]. The gene symbols of the CRGs were shown in the Supplementary Table 2.

### Differential expression analysis and protein–protein interaction (PPI) network analysis of CRGs

The data was rectified using log2 conversion prior to differential expression analysis. Subsequently, the expression data of CRGs was acquired from training sets and the “limma” package was employed to find differentially expressed CRGs between patients with three kinds of primary cardiomyopathy and healthy people, thereby obtaining differential CRGs for DCM, HCM and ARVC, respectively. The criterion of differential CRGs was *P*-value less than 0.05. After that, we employed the “pheatmap” package and “pheatmap” package to generate differential CRGs heatmaps and boxplots. Then, the intersection of differential CRGs of the three diseases was employed to obtain differential CRGs shared by three kinds of primary cardiomyopathy. Finally, the PPI network analysis of the CRGs shared by three kinds of primary cardiomyopathy was conducted by using STRING database to explore whether there was any interaction between these CRGs. In addition, the “RCircos” package was employed to exhibit the chromosomal location of CRGs.

### Unsupervised clustering for three kinds of primary cardiomyopathy patients

The unsupervised clustering analysis of three kinds of primary cardiomyopathy groups was conducted by employing the R package “ConsensusClusterPlus”. The molecular subtype clusters were distinguished according to the expression level of CRGs. The optimum amounts of clusters for each primary cardiomyopathy were assessed on the basis of consensus clustering matrix, cumulative distribution function (CDF) curves, CDF delta area curves and consensus clustering score.

### Single-gene gene set enrichment analysis (GSEA)

We identified biological functions correlated to the individual CRG in this study by single-gene GSEA. In the first step, we calculated the relevance between all other genes in the whole gene set and the shared differential CRGs, and sorted the gene set based on the relevance. We then employed the Gene Ontology (GO) and Kyoto Encyclopedia of Genes and Genomes (KEGG) data files as references to assess the degree of enrichment of each gene set on different biological functions to find the biological functions most strongly associated with individual CRGs. Finally, the enrichment results of each single gene were visualized separately. These analysis processes and visualization processes were carried out with the support of packages “limma”, “org.Hs.eg.db”, “clusterProfiler” and “enrichplot”.

### Gene set variation analysis (GSVA)

We identified biological functions correlated to the three kinds of primary cardiomyopathy or different molecular subtypes clusters in this study by GSVA. In common with GESA, GSVA also references GO and KEGG data files. With the support of the R software package “reshape2”, “limma”, “GSEABase” and “GSVA”, GSVA analysis was performed to identify the differential biological functions between the three kinds of primary cardiomyopathy groups and the control group or between clusters of different molecular subtypes. Differential biological functions were determined by absolute t value of GSVA score greater than 2.

### Immune cell infiltration analysis

With the support of R package “preprocessCore”, we calculated the abundance and proportion of immune cell infiltration in each sample based on CIBERSORT algorithm and gene expression profile of each sample, thus finding the differences of immune infiltration between patients with three kinds of primary cardiomyopathy and healthy people, as well as the differences of immune infiltration between different molecular subtype clusters in each kind of primary cardiomyopathy. A total of 22 of the most common types of immune cells were included in the CIBERSORT algorithm analysis.

### Establishment and estimation of diagnostic model and molecular subtype identification model

The least absolute shrinkage and selection operator (LASSO) regression was carried out by employing “glmnet” package to estimate the value of differential CRGs shared by three kinds of primary cardiomyopathy in the disease diagnosis, thereby obtaining the key CRGs (K-CRGs) with high diagnostic value. After that, R packages “caret” was employed to build three machine learning models on the basis of K-CRGs, including random forest model (RF), support vector machine model (SVM) and generalized linear model (GLM). Finally, the diagnosis and molecular subtype identification performed by these models was estimated via cumulative residual distribution curves, residual boxplots and receiver operating characteristic (ROC) curves.

### Establishment of the nomogram

We employed “rms” package to produce the K-CRGs-based nomograms for the diagnosis of three kinds of primary cardiomyopathy. In the nomogram, each K-CRGs was assigned a different score depending on its expression level, and the sum of all K-CRGs scores could predict the risk of three kinds of primary cardiomyopathy. Finally, we performed calibration curve and decision curve analysis (DCA) to estimate the accuracy of these nomograms in diagnosing three kinds of primary cardiomyopathy.

### Construction of ceRNA network

The miRNA that may regulate the K-CRGs was forecasted according to the miRanda, miRDB and TargetScan databases. At the same time, lncRNA that may regulate the miRNA was forecasted according to the lncBase and mircode databases. The ceRNA network of mRNA-miRNA-lncRNA interaction was built by employing the Cytoscape tool.

### Prediction of targeted drugs

We searched the DSigDB database for drugs of regulating K-CRGs and visualized the result by employing the Cytoscape tool.

### Molecular docking

First, we employed the PubChem database to obtain the 2D structures of targeted drugs. Then, we employed the Chem3D tool to transform drug 2D structure into 3D. After that, we employed the PDB database and UniProt database to obtain the 3D structure of K-CRGs. Subsequently, we employed the Autodock Tool to position active pocket of K-CRGs. In the end, we used the Autodock Vina tool to conduct molecular docking, thereby forecasting the binding sites and binding strength between K-CRGs and drugs.

### Materials and reagents

The antibodies of FDX1 (12592-1-AP), SLC31A1 (67221-1-Ig), LIAS (11577-1-AP), POLD1 (15646-1-AP) and HSP70 (10995-1-AP) were purchased from Proteintech Group, USA. The antibody of MAP2K1 (4694) was purchased from Cell Signaling Technology, USA. The copper chloride (CuCl_2_) (C3279) and glutathione (GSH) (G6013) were obtained from Sigma Aldrich, USA. The Dulbecco’s Modified Eagle’s Medium (DMEM) (21331046), fetal bovine serum (FBS) (10100147C), and penicillin-streptomycin (15140122) were obtained from Gibco, USA. The siRNAs of FDX1 (107318), SLC31A1 (10548) and MAP2K1 (142322) were bought from Ambion, USA. The transfection reagent Lipofectamine RNAiMAX (13778075) was purchased from Invitrogen, USA. The reverse transcription reagents (11141ES60) and amplification reagents (11202ES08) for real time quantitative polymerase chain reaction (RT-qPCR) were purchased from Yeasen Biotechnology, China.

### Cell culture and model establishment

The AC16 cell is a myocardial cell line derived from human ventricular tissue and has the ability of proliferation *in vitro* [20]. AC16 cells have the same nuclear DNA and mitochondrial DNA as ventricular tissue derived primary cardiomyocytes, and retain the mitochondrial respiratory function of primary cardiomyocytes [21]. Previous literature has shown that AC16 cells are suitable for *in vitro* experimental studies of a variety of primary cardiomyopathy in humans, especially for studying the pathogenesis of primary cardiomyopathy related to mitochondrial function [22, 23]. The data in the bioinformatics analysis part of our study were derived from human ventricular tissue samples, and cuproptosis was closely related to mitochondrial function. Therefore, the *in vitro* experimental part of our study selected AC16 cell lines as tool cells to verify the role and expression level changes of K-CRGs in cuproptosis of cardiomyocytes. The AC16 cells were provided by Merck-Millipore, USA. The AC16 cells were cultured in DMEM supplemented with 12.5% FBS and 1% penicillin-streptomycin. The incubator conditions are set to: 37°C and 5% CO_2_. The method of establishing cell cuproptosis model in this study was referred to previous research [24]: AC16 cells were treated with different concentrations of CuCl_2_ for 24 hours and CCK-8 assay was performed. The concentration of CuCl_2_, which could significantly reduce cells viability, was selected as the intervention concentration for subsequent experiments. The GSH is a common copper chelator that binds intracellular copper and could be employed as the cuproptosis inhibitor.

### Transfection of siRNA

We diluted the siRNA and transfection reagents by employing serum-reducing medium at the suggested concentrations in the specification. After that, the diluted siRNA solution and the transfection reagent solution were mixed. The AC16 cells were then cultured in the mixture for 48 hours. Finally, the RT-qPCR experiment was employed to verify whether the gene was successfully knocked down.

### CCK-8 cell proliferation assay

AC16 cells were cultured in 96-well plates to a density of approximately 80 percent. The CuCl_2_ was then added to each well and cultured for 24 h according to the following concentration gradients: 5 μM, 10 μM, 25 μM, 50 μM, 75 μM, and 100 μM. After that, 10 μL CCK8 solution was added to each well for 2 hours and absorbance was measured at 450 nm.

### Western blot

First, we employed gel electrophoresis technology to isolate proteins extracted from AC16 cells. Then, we employed protein transfer technology to transfer the isolated proteins from the gel to the PVDF membrane. After that, we employed 5% bovine serum albumin to block the protein on the PVDF membrane for one hour. Subsequently, the PVDF membrane was incubated with FDX1, SLC31A1, MAP2K1, LIAS, POLD1, HSP70 and DLAT primary antibodies at 4°C overnight. On the second day, the PVDF membrane was incubated with the corresponding secondary antibodies at room temperature for 1 hour. Finally, the chemiluminescence reaction and blot imaging were performed on the PVDF membrane.

### RT-qPCR

The experimental parameters of RT-qPCR were set according to the specification. The parameters of the reverse transcription were set to: 25°C for five minutes, 55°C for 15 minutes, 85°C for five minutes. The parameters of the amplification were set to: pre-denaturation for five minutes at 95°C, followed by 40 cycles at 95°C for 10 seconds and 60°C for 30 seconds. The relative expression level of the mRNA of the target gene was assessed by the ΔΔCt method. The Supplementary Table 3 exhibited the primer sequences (primer synthesis was carried out by Generay Biotech, China).

### Immunofluorescence

The AC16 cells seeded on glass coverslips were fixed with paraformaldehyde for 30 minutes, and then the cells were blocked in 3% goat serum for one hour. Next, the cells were incubated in the primary antibodies of FDX1, MAP2K1 and SLC31A1 overnight at 4°C. After that, the cells were incubated in cyanine 3 (Cy3) coupled secondary antibodies for one hour under dark conditions. Finally, the cell nuclei were stained with 4′,6-diamidino2-phenylindole (DAPI) staining solution.

### Statistical analysis

The R 4.2.1 software was employed to perform bioinformatics analysis. The GraphPad Prism 7.0 software was employed to perform *in vitro* experiment data analysis. Three or more sets of data were compared by employing One-way ANOVA. The statistically significant difference was defined as *P*-value less than 0.05 (*P* < 0.05).

### Data availability

All data of this study is involved in the article and supplementary materials, additional data could be acquired by connecting corresponding authors.

## RESULTS

### Identification of differential CRGs in training sets

**Figure 1:**
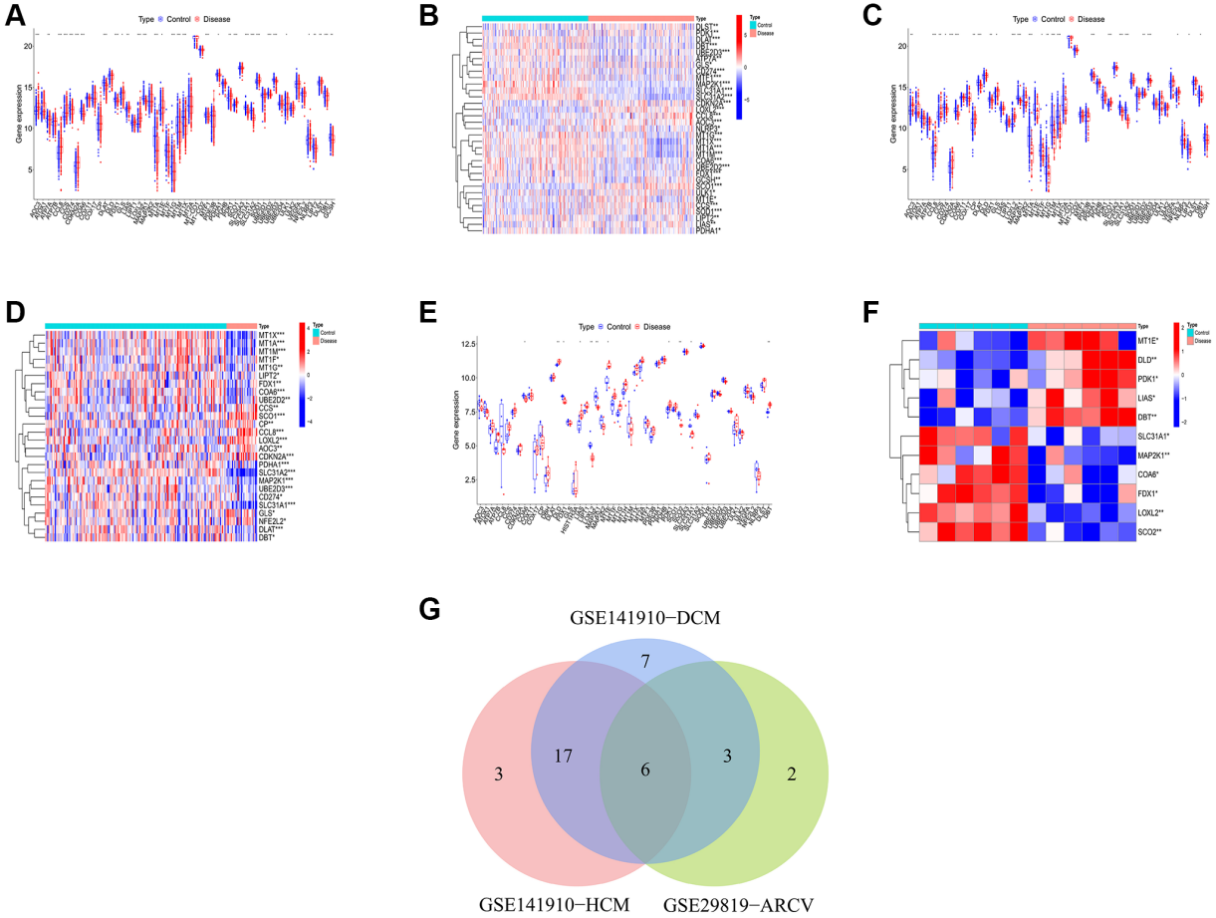
**Identification of shared differential CRGs.** (**A**, **B**) The box plot and heatmap of differential CRGs in DCM. (**C**, **D**) The box plot and heatmap of differential CRGs in HCM. (**E**, **F**) The box plot and heatmap of differential CRGs in ARVC. (**G**) The Venn diagram of differential CRGs shared by the three kinds of primary cardiomyopathy.

According to the previous research, a total of 59 CRGs have been identified [16–19]. Then, we conducted differential expression analysis of CRGs between primary myocardiopathy group and control group in training sets. The results indicated that 33 differential CRGs were identified in DCM, 26 in HCM and 11 in ARVC (Figure 1A–1F). Next, through the intersection of the differential CRGs of three diseases, six shared differential CRGs were obtained (Figure 1G). Finally, we checked the changes in the expression trends of these six genes, and found that there were four genes whose expression trends changed consistently in the three diseases, they were all downregulated in the disease group. Therefore, we ultimately identified four genes as differential CRGs shared by the three kinds of primary cardiomyopathy: COA6, FDX1, MAP2K1, and SLC31A1. The result of PPI network indicated that these four genes do not interact with each other (Supplementary Figure 2).

### Functional analysis of the shared differential CRGs

**Figure 2:**
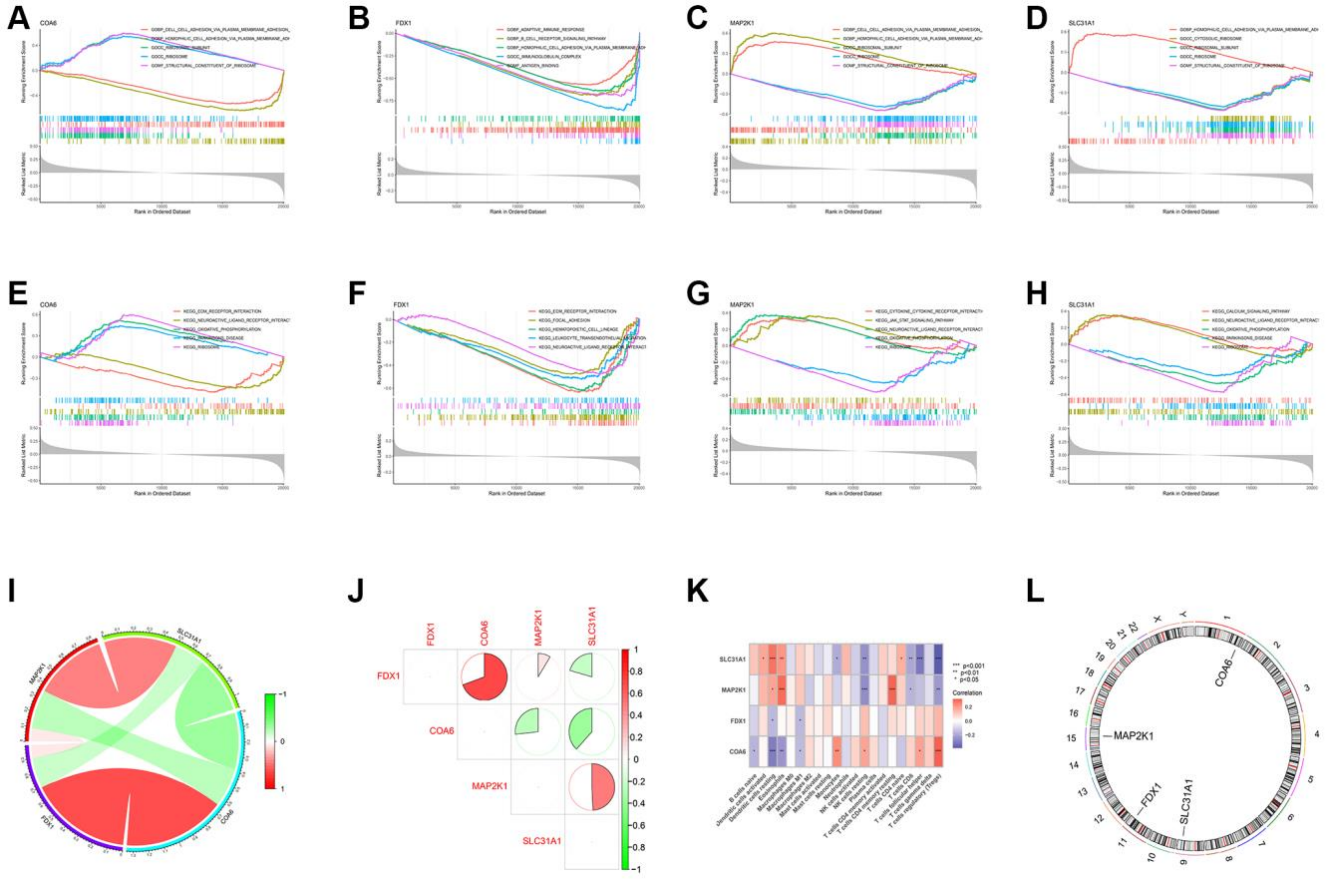
**Functional analysis of four shared differential CRGs.** (**A**–**D**) Single-gene GSEA GO analysis of four shared differential CRGs. (**E**–**H**) Single-gene GSEA KEGG analysis of four shared differential CRGs. (**I**, **J**) The correlation analysis of four shared differential CRGs. (**K**) The correlation analysis of four shared differential CRGs and immune cells. (**L**) The position of four shared differential CRGs on chromosome.

**Figure 3:**
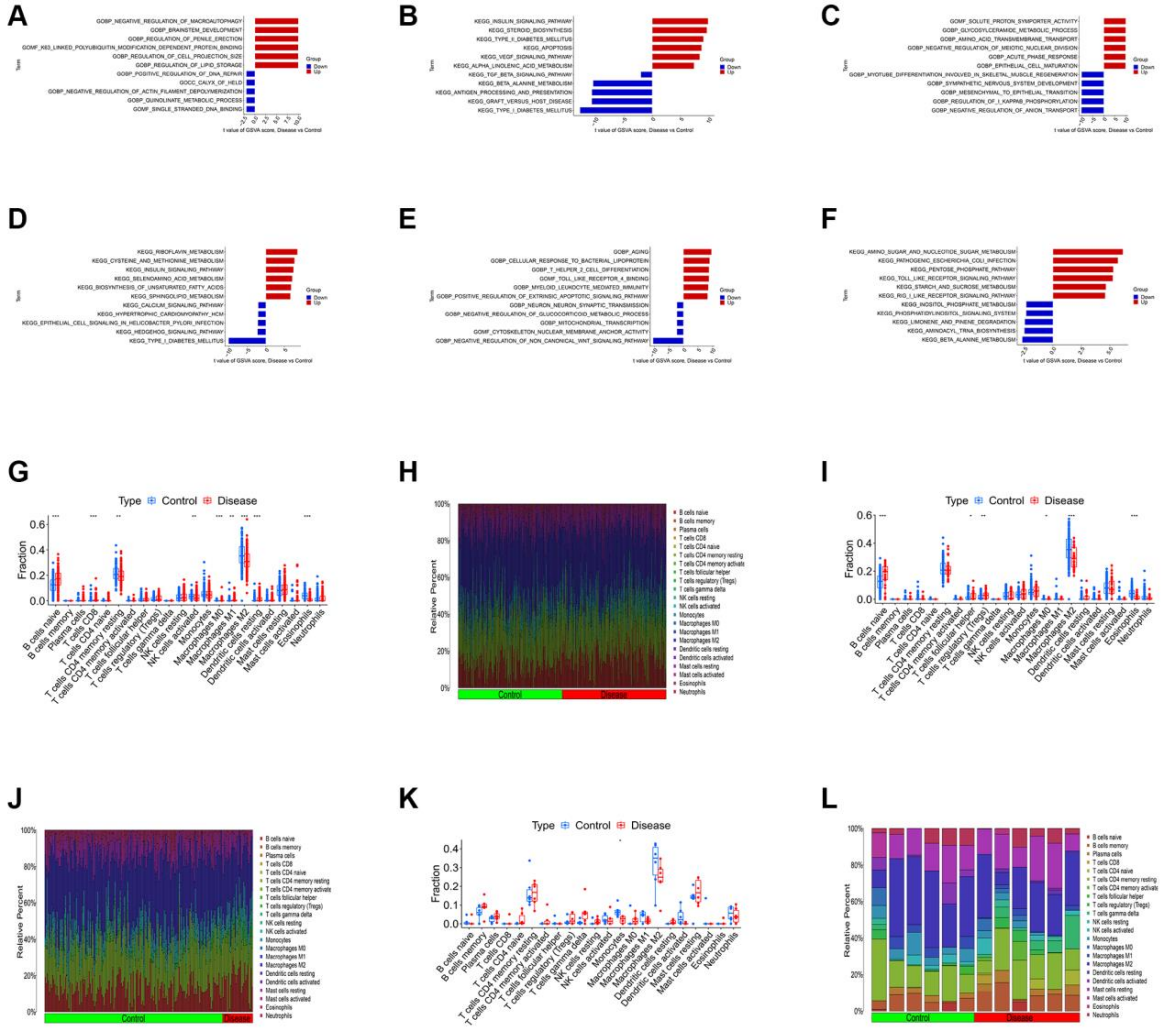
**Differential function analysis between three kinds of primary cardiomyopathy group and control group.** (**A**, **B**) The GSVA-GO analysis and GSVA-KEGG analysis for DCM. (**C**, **D**) The GSVA-GO analysis and GSVA-KEGG analysis for HCM. (**E**, **F**) The GSVA-GO analysis and GSVA-KEGG analysis for ARVC. (**G**, **H**) The box plot and bar plot of infiltrated immune cells in DCM. (**I**, **J**) The box plot and bar plot of infiltrated immune cells in HCM. (**K**, **L**) The box plot and bar plot of infiltrated immune cells in ARVC.

We first conducted the single-gene GSEA to detect the biological functions associated with the four shared differential CRGs. The results indicated that the shared CRGs were related to multiple immunoinflammatory processes, such as cell adhesion, adaptive immune response, B cell receptor signaling pathway and cytokine-cytokine receptor interaction (Figure 2A–2H). Some other recent studies have also reported the correlation between CRGs and immune inflammation, suggesting that the process of cuproptosis may be accompanied by changes in the immune inflammatory system, and the exact mechanism requires further investigation in the future [25, 26]. Considering the relevance between these shared differential CRGs and immuno-inflammatory response, we studied the relevance between the levels of shared differential CRGs and the levels of immune cells infiltration. The results indicated that levels of the shared differential CRGs were not only correlated with each other, but also associated with the levels of multiple immune cells infiltration (Figure 2I–2K). Moreover, the chromosome positions of the shared CRGs were presented in the Figure 2L. To further research the role of cuproptosis and immuno-inflammatory response in the three kinds of primary cardiomyopathy, GSVA analysis and immune infiltration analysis were conducted. The results of GSVA indicated that biological processes such as cell death, mitochondria-related function and immune inflammation were significantly different between the three kinds of primary cardiomyopathy groups and the control groups, including apoptosis, transcription process of mitochondria, IkappaB phosphorylation, T helper 2 cell differentiation and Toll-like receptor signaling pathway (Figure 3A–3F). The results of immune infiltration analysis also indicated that there were obviously differences in the levels of multiple immune cells infiltration between the three kinds of primary cardiomyopathy groups and the control groups (Figure 3G–3L).

### Construction of diagnostic model based on K-CRGs

**Figure 4:**
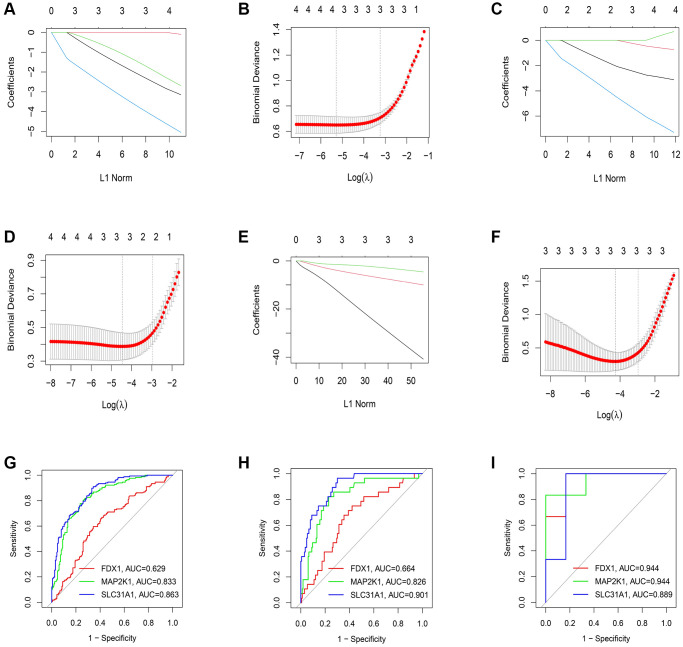
**Estimation of the diagnosis efficacy of four shared differential CRGs.** (**A**, **B**) The LASSO coefficient and most appropriate lambda value of four shared differential CRGs in DCM. (**C**, **D**) The LASSO coefficient and most appropriate lambda value of four shared differential CRGs in HCM. (**E**, **F**) The LASSO coefficient and most appropriate lambda value of four shared differential CRGs in ARVC. (**G**–**I**) Estimation of the diagnosis efficacy of three K-CRGs via ROC analysis in DCM, HCM and ARVC.

**Figure 5:**
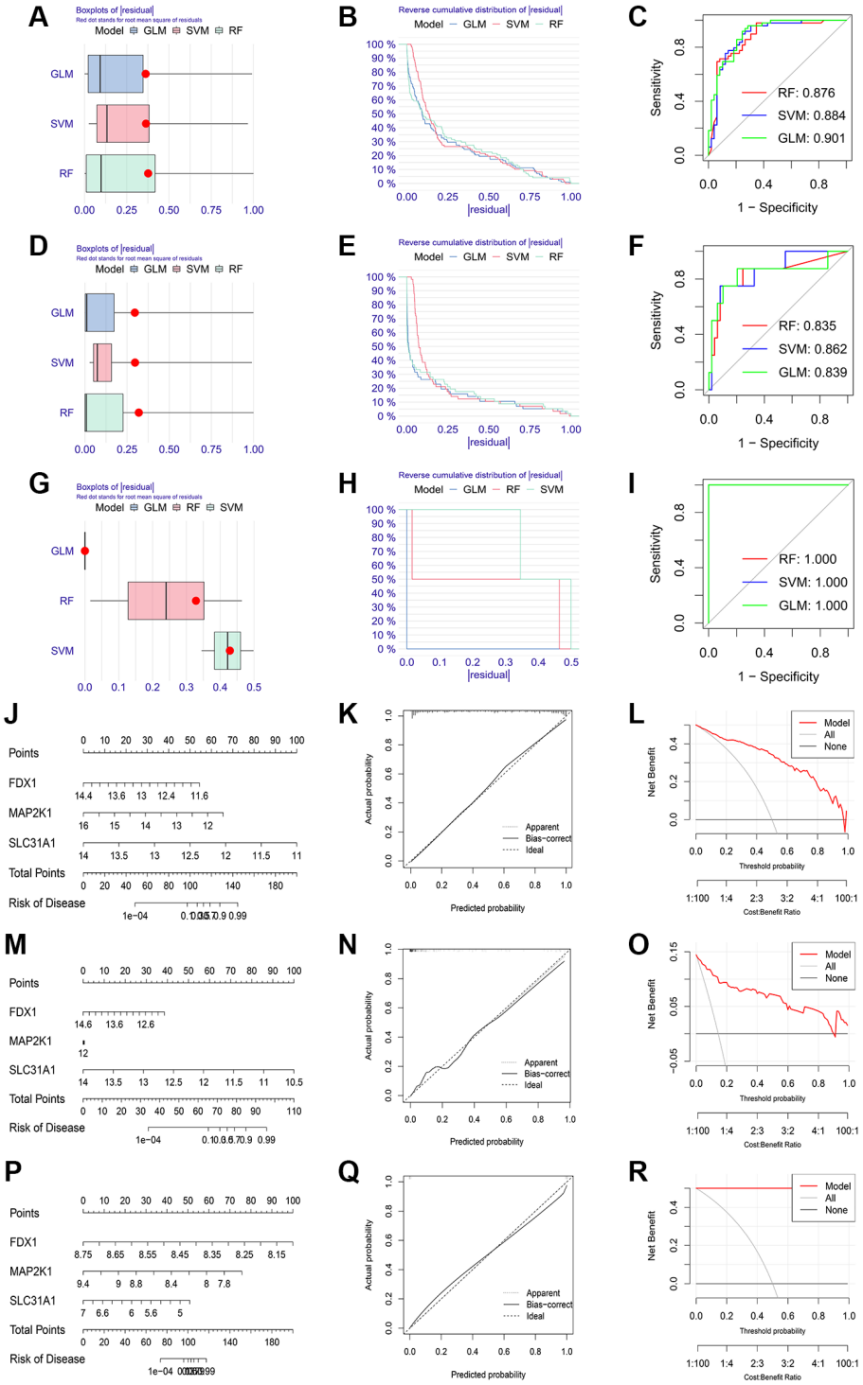
**Establishment of diagnosis model on the basis of three K-CRGs.** (**A**–**C**) The residual boxplots, cumulative residual distribution curves and ROC curves of three machine learning algorithms in DCM. (**D**–**F**) The residual boxplots, cumulative residual distribution curves and ROC curves of three machine learning algorithms in HCM. (**G**–**I**) The residual boxplots, cumulative residual distribution curves and ROC curves of three machine learning algorithms in ARVC. (**J**) The nomogram of DCM. (**K**, **L**) The calibration curves and DCA of the DCM nomogram. (**M**) The nomogram of HCM. (**N**, **O**) The calibration curves and DCA of the HCM nomogram. (**P**) The nomogram of ARVC. (**Q**, **R**) The calibration curves and DCA of the ARVC nomogram.

We conducted LASSO regression to assess the performance of four shared differential CRGs in the diagnosis of three kinds of primary cardiomyopathy and three K-CRGs with the greatest diagnostic performance were chosen (Figure 4A–4F). Then, we estimated the efficacy of the three K-CRGs in the diagnosis of three kinds of primary cardiomyopathy, and the results indicated that some genes had well diagnostic performance (Figure 4G–4I). After that, we built three machine learning models (RF, SVM and GLM) on the basis of three K-CRGs. We employed three assessment methods (residual boxplots, cumulative residual distribution curves and ROC curves) to assess the diagnostic performance of three models. The results indicated that all the three models exhibited the outstanding diagnostic value (Figure 5A–5I). Lastly, we built the nomograms on the basis of three K-CRGs to predict the risk of primary cardiomyopathy and validated the predictive value of the nomograms by employing two assessment methods (calibration curves and DCA). The results indicated that the predictive value of the nomograms was excellent (Figure 5J–5R).

### Identification of cuproptosis-related molecular subtype based on K-CRGs

**Figure 6:**
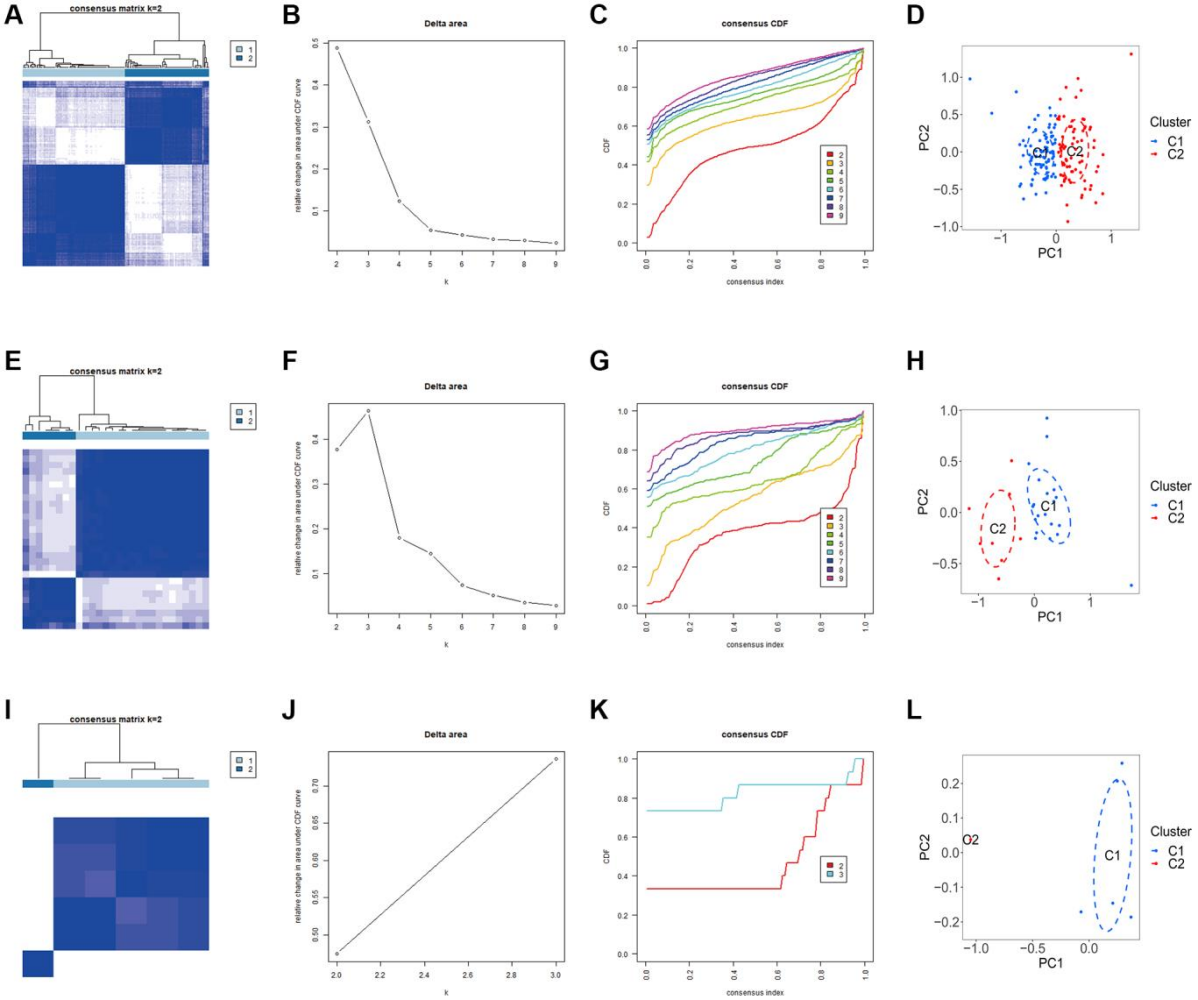
**Identification of molecular subtypes clusters in the three kinds of primary cardiomyopathy.** (**A**–**D**) The consensus clustering matrix, CDF curves, CDF delta area curves and PCA of DCM. (**E**–**H**) The consensus clustering matrix, CDF curves, CDF delta area curves and PCA of HCM. (**I**–**L**) The consensus clustering matrix, CDF curves, CDF delta area curves and PCA of ARVC.

**Figure 7:**
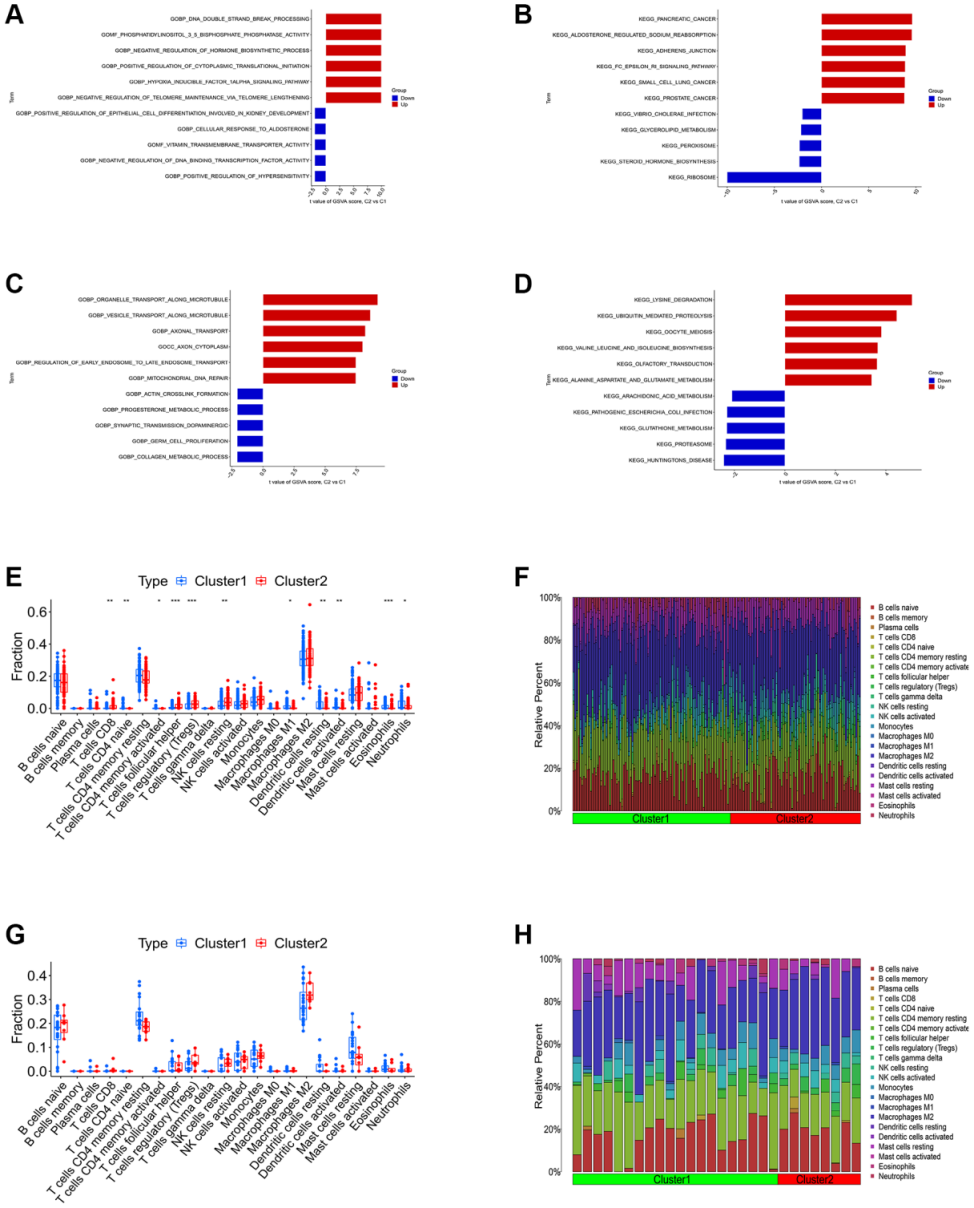
**Differential function analysis between different molecular subtypes clusters.** (**A**, **B**) The GSVA-GO analysis and GSVA-KEGG analysis for two cluster of DCM. (**C**, **D**) The GSVA-GO analysis and GSVA-KEGG analysis for two cluster of HCM. (**E**, **F**) The box plot and bar plot showing the differences in infiltrated immune cells between two clusters of DCM. (**G**, **H**) The box plot and bar plot showing the differences in infiltrated immune cells between two clusters of HCM.

**Figure 8:**
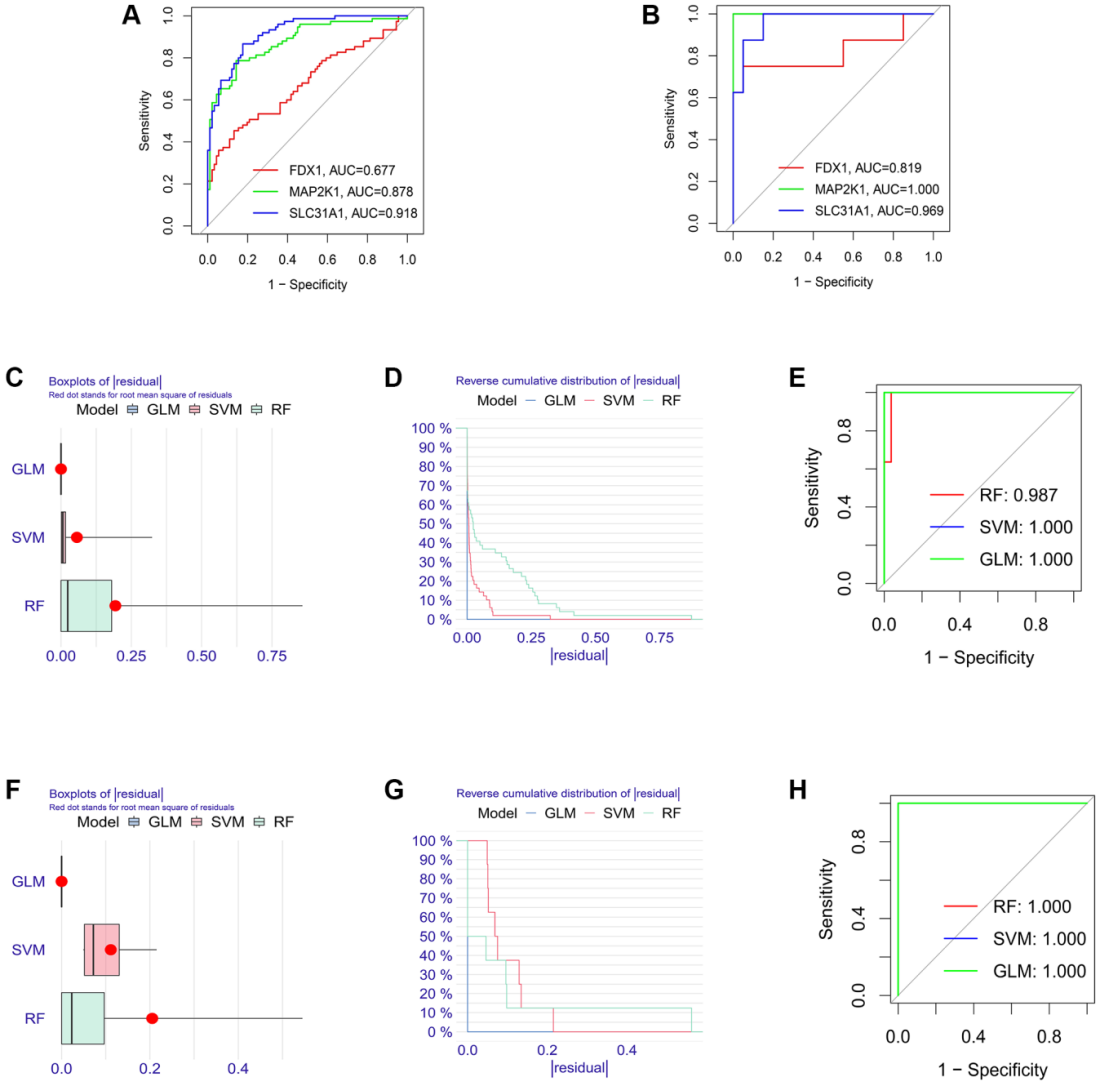
**Establishment of the molecular subtype identification efficacy of three K-CRGs.** (**A**, **B**) Establishment of the molecular subtype identification efficacy of three K-CRGs via ROC analysis in DCM and HCM. (**C**–**E**) The residual boxplots, cumulative residual distribution curves and ROC curves of three machine learning algorithms for DCM molecular subtype identification. (**F**–**H**) The residual boxplots, cumulative residual distribution curves and ROC curves of three machine learning algorithms for HCM molecular subtype identification.

We carried out the unsupervised clustering analysis on the training sets according to the expression levels of three K-CRGs to identify the molecular subtypes of three kinds of primary cardiomyopathy. We employed four assessment methods (consensus clustering matrix, CDF curves, CDF delta area curves and consensus clustering score) to determine the most appropriate numbers of molecular subtype clusters for each primary cardiomyopathy. The results indicated that the most appropriate numbers of molecular subtype clusters for each primary cardiomyopathy was two (Figure 6A–6C, 6E–6G, 6I–6K, Supplementary Figures 3A–3O and 4A–4U). Therefore, we divided DCM patients, HCM patients and ARVC patients into two molecular subtype clusters respectively (cluster 1 and cluster 2). The results of PCA indicated that in each disease, the two clusters were obviously split (Figure 6D, 6H, 6L). Since the K-CRGs are correlated with cuproptosis and immuno-inflammatory response, we then tried to determine whether there were differences in cell death, mitochondria-related function and immune inflammation between the two molecular subtype clusters in each disease (since there was only one sample in cluster 2 of disease ARVC, subsequent analysis was only carried out for disease DCM and disease HCM). The results of GSVA indicated that significant differences in numerous biological functions related to cell death, mitochondrion and immune inflammation, such as apoptosis, cell proliferation, mitochondrial DNA repair, antigen-stimulated inflammatory response and macrophage colony stimulating factor response (Figure 7A–7D). In addition, the immune analysis results also indicated that there were differences in immune cells infiltration among different clusters (Figure 7E–7H). Finally, we evaluated the efficacy of K-CRGs in identifying molecular subtype clusters of each primary cardiomyopathy. The results of ROC analysis indicated that partial single gene exhibited good performance in identifying molecular subtypes of primary cardiomyopathy, and the machine learning models on the basis of three K-CRGs exhibited better performance (Figure 8A–8H).

### Verification of the diagnostic value of K-CRGs

**Figure 9:**
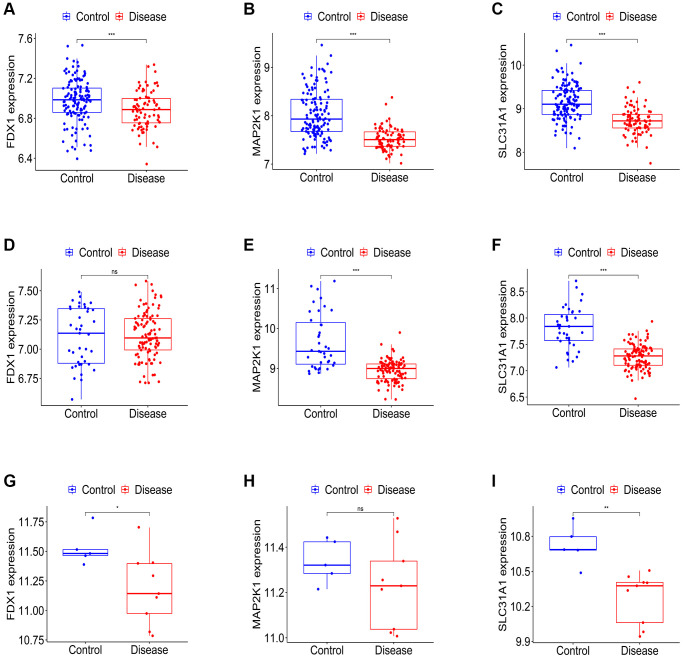
**Establishment of the expression levels of three K-CRGs in validation set.** (**A**–**C**) The expression levels of three K-CRGs in DCM validation set. (**D**–**F**) The expression levels of three K-CRGs in HCM validation set. (**G**–**I**) The expression levels of three K-CRGs in ARVC validation set.

**Figure 10:**
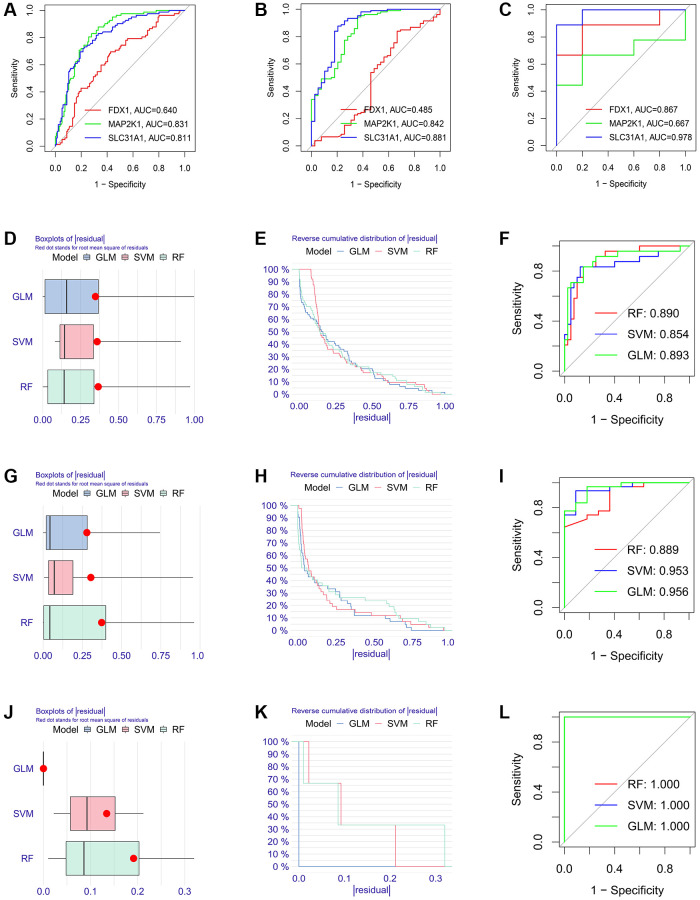
**Estimation of the diagnosis efficacy of three K-CRGs in validation set.** (**A**–**C**) Estimation of the diagnosis efficacy of three K-CRGs for DCM, HCM and ARVC via ROC analysis in validation set. (**D**–**F**) The residual boxplots, cumulative residual distribution curves and ROC curves of three machine learning algorithms in DCM validation set. (**G**–**I**) The residual boxplots, cumulative residual distribution curves and ROC curves of three machine learning algorithms in HCM validation set. (**J**–**L**) The residual boxplots, cumulative residual distribution curves and ROC curves of three machine learning algorithms in ARVC validation set.

**Figure 11:**
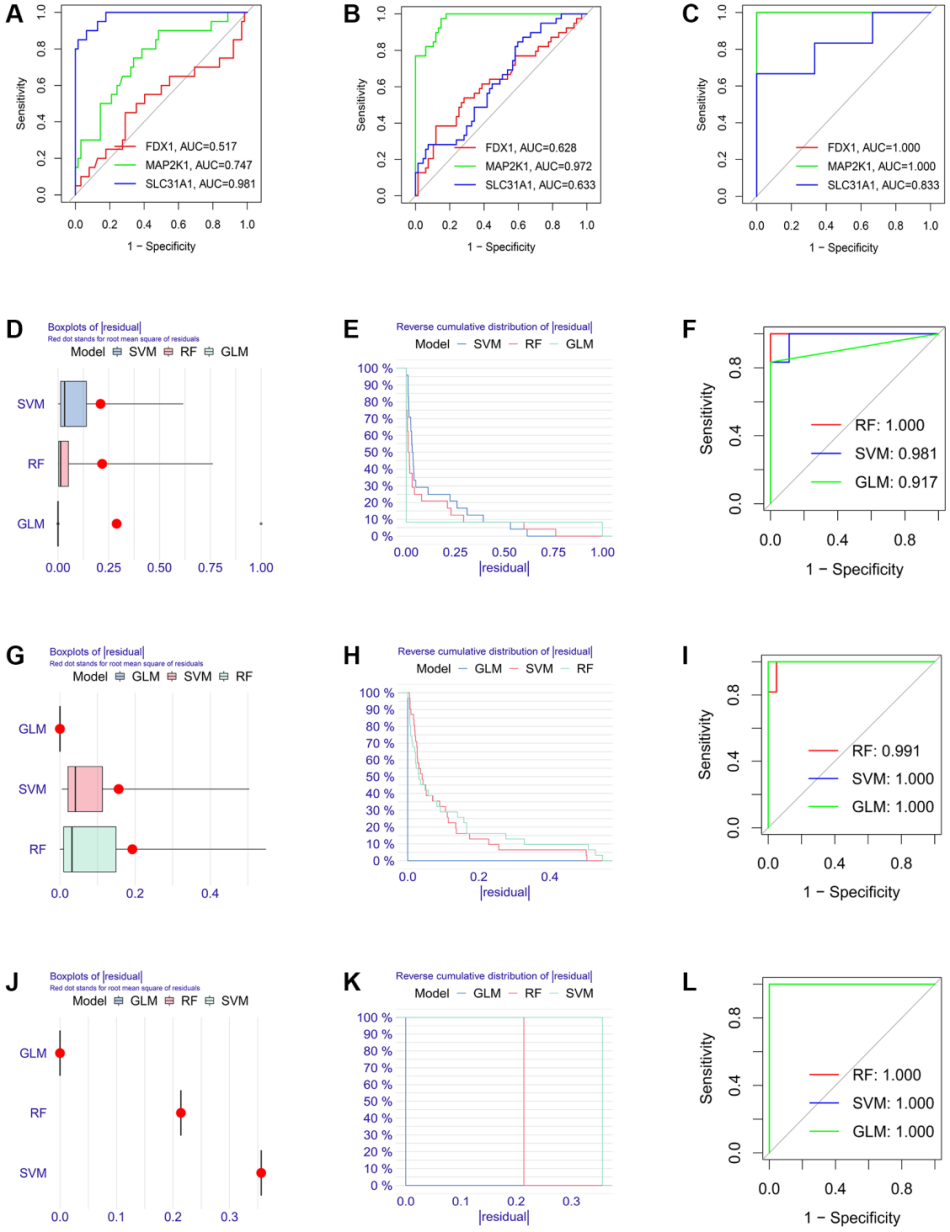
**Establishment of the molecular subtype identification efficacy of three K-CRGs in validation set.** (**A**–**C**) Estimation of the molecular subtype identification efficacy of three K-CRGs for DCM, HCM and ARVC via ROC analysis in validation set. (**D**–**F**) The residual boxplots, cumulative residual distribution curves and ROC curves of three machine learning algorithms for DCM molecular subtype identification in validation set. (**G**–**I**) The residual boxplots, cumulative residual distribution curves and ROC curves of three machine learning algorithms for HCM molecular subtype identification in validation set. (**J**–**L**) The residual boxplots, cumulative residual distribution curves and ROC curves of three machine learning algorithms for ARVC molecular subtype identification in validation set.

Firstly, the expression levels of three K-CRGs were validated in the independent validation set of three kinds of primary cardiomyopathy. The results indicated that the expression trends of these K-CRGs in independent validation sets were concordant with their expression trends in training sets of each primary cardiomyopathy (Figure 9A–9I). Then, the diagnostic efficacy of three K-CRGs was assessed in independent validation sets. The results indicated that partial single gene exhibited good diagnostic value (Figure 10A–10C), and the machine learning models on the basis of three K-CRGs exhibited better value (Figure 10D–10L). Next, the molecular subtype identification efficacy of the K-CRGs was assessed in an independent validation set. The results indicated that the most appropriate numbers of molecular subtype clusters for each primary cardiomyopathy was two (Supplementary Figures 5A–5U and 6A–6X). In the end, the results of ROC analysis indicated that partial single gene exhibited good performance in identifying molecular subtypes of primary cardiomyopathy (Figure 11A–11C), and the machine learning models on the basis of three K-CRGs exhibited better performance (Figure 11D–11L).

### Regulatory molecular prediction of K-CRGs

**Figure 12:**
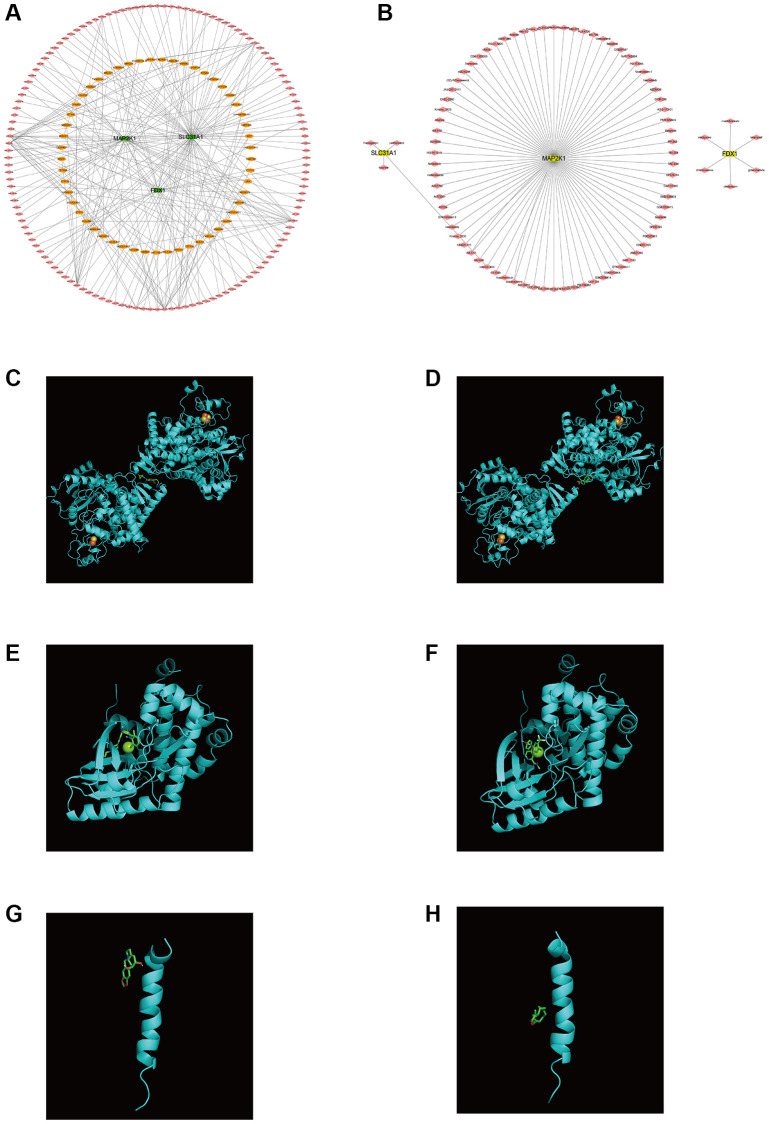
**Regulatory molecular prediction of K-CRGs.** (**A**) The ceRNA network of K-CRGs. (**B**) The targeted drug network of K-CRGs. (**C**) Forecast of combinative location between FDX1 and latamoxef (their combined free energy is -7.7). (**D**) Forecast of combinative location between FDX1 and glibenclamide (their combined free energy is -7.5). (**E**) Forecast of combinative location between MAP2K1 and dasatinib (their combined free energy is -9.8). (**F**) Forecast of combinative location between MAP2K1 and sorafenib (their combined free energy is -9.7). (**G**) Forecast of combinative location between SLC31A1 and lycorine (their combined free energy is -5.5). (**H**) Forecast of combinative location between SLC31A1 and parthenolide (their combined free energy is -5.1).

Firstly, the ceRNA network was built on the basis of three K-CRGs. The network contained 188 nodes (three mRNA, 132 miRNAs and 53 lncRNAs) and 224 edges (Figure 12A). The interactive relation of each mRNA, miRNA and lncRNA was exhibited in Supplementary Table 4. Then, the targeted drugs of three K-CRGs were forecasted by employing the DSigDB database, and in all 83 drugs were acquired. Of these drugs, 74 drugs possibly regulate MAP2K1 gene, six drugs possibly regulate FDX1 gene and four drugs possibly regulate SLC31A1 gene (Figure 12B). In the end, the molecular docking was conducted to forecast the binding sites and binding strength of these K-CRGs and drugs. In Figure 12C–12H, we visualized the two drugs that bind most firmly to each K-CRGs.

### CuCl_2_ could induce cuproptosis in AC16 cells and change the expression levels of K-CRGs

**Figure 13:**
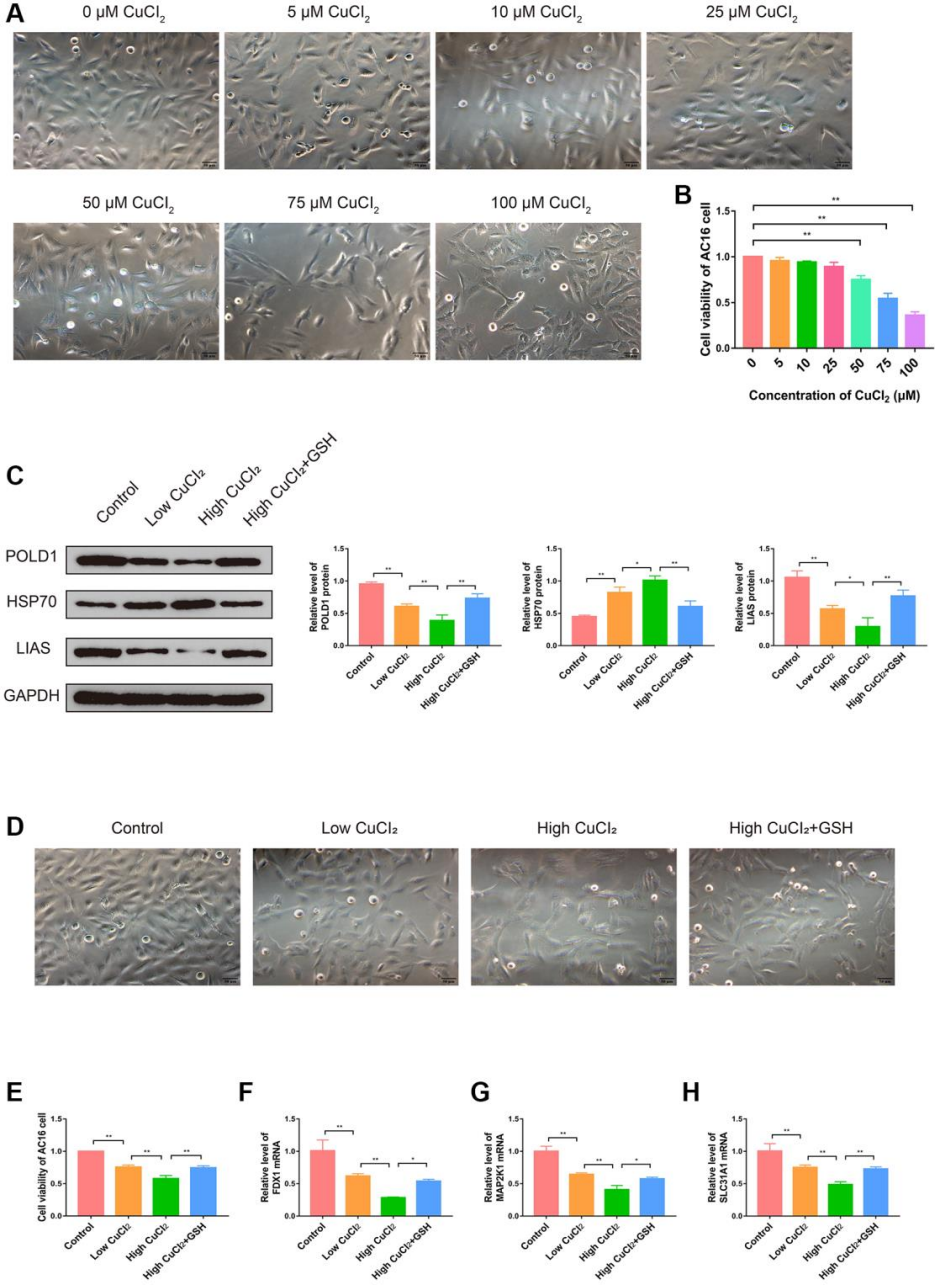
**Effects of CuCl_2_ on morphology, viability and cuproptosis degree of AC16 cells.** (**A**) The effects of CuCl_2_ on AC16 cells morphology. (**B**) The effects of CuCl_2_ on AC16 cells viability. (**C**) The protein expression levels of POLD1, HSP70 and LIAS in AC16 cells (*n* = 3). (**D**) The effects of GSH on AC16 cells morphology. (**E**) The effects of GSH on AC16 cells viability. (**F**–**H**) The mRNA expression levels of three K-CRGs in AC16 cells. (^*^P < 0.05, ^**^P < 0.01).

**Figure 14:**
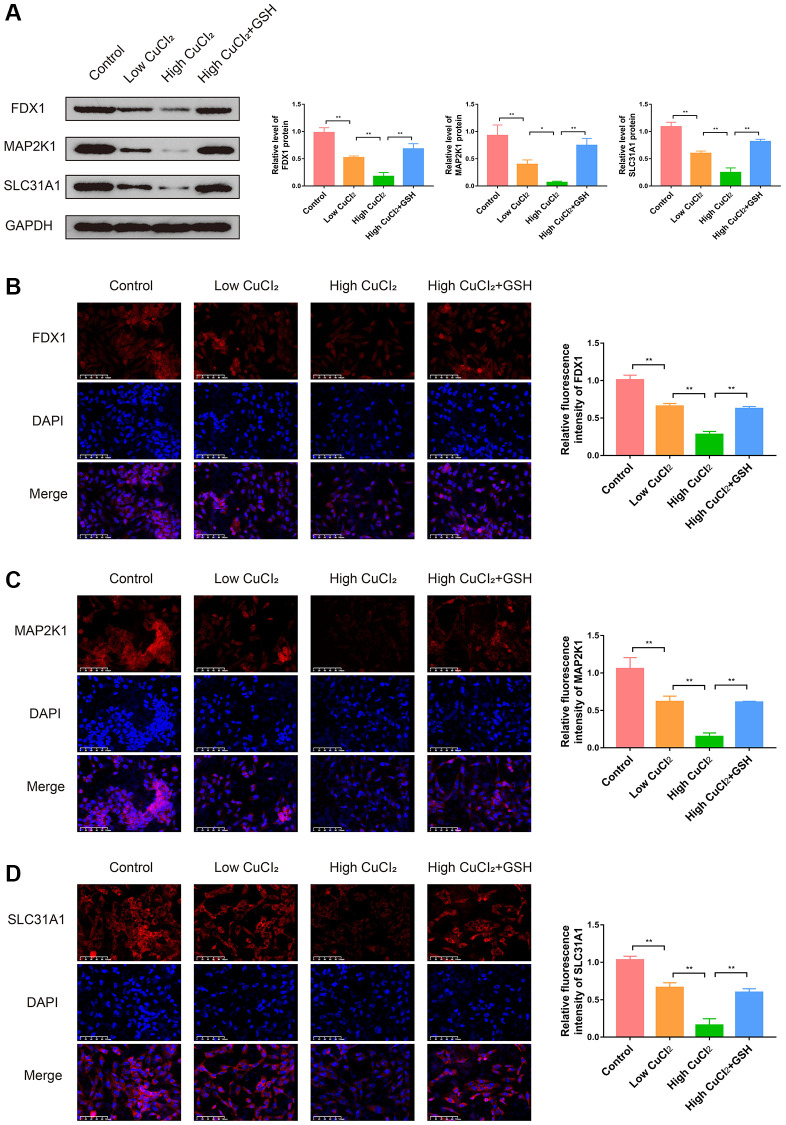
**Effects of CuCl_2_ on the expression levels of three K-CRGs in AC16 cells.** (**A**) The protein expression levels of three K-CRGs in AC16 cells (*n* = 3). (**B**–**D**) Typical immunofluorescence images and quantitative analysis of three K-CRGs in AC16 cells (*n* = 3). (^*^P < 0.05, ^**^P < 0.01).

In order to investigate the role of cuproptosis in cardiomyopathy and the changes in the expression levels of three K-CRGs, we conducted *in vitro* experiments. Firstly, the suitable CuCl_2_ concentration of construction the cuproptosis model of AC16 cells was selected via CCK-8 assay. The results indicated that CuCl_2_ could lead to cell morphological changes and death when the concentration reached 50 μM (Figure 13A, 13B). Therefore, 50 μM concentration of CuCl_2_ was selected as low dose and 75 μM concentration of CuCl_2_ was selected as high dose for subsequent experiments. Next, we verified whether the CuCl_2_-induced cell death mode was cuproptosis. The AC16 cells were treated with CuCl_2_ or CuCl_2_ combined with GSH, and the expression levels of cuproptosis marker proteins (LIAS, POLD1 and HSP70) in the cells were detected. The results indicated that with the increase of the concentration of CuCl_2_, the degree of cell cuproptosis gradually increased (specifically manifested as the up-regulation of HSP70 protein, the down-regulation of LIAS and POLD1 protein), and the degree of cell cuproptosis decreased after the GSH was employed to bind CuCl_2_ (Figure 13C). In addition, the morphological experiment and CCK-8 assay results also showed that cell morphological changes and death were suppressed when the GSH was employed, further indicating that cuproptosis might occur in AC16 cells (Figure 13D, 13E). Then, we detected the mRNA expression levels of three K-CRGs in AC16 cells. The results indicated that the mRNA expression levels of three K-CRGs in the CuCl_2_ group were significantly down-regulated compared with the control group, and these expression trend changes were significantly weakened in the GSH combined with CuCl_2_ group (Figure 13F–13H). Accordant results were acquired in subsequent experiments of western blot and immunofluorescence, the protein expression levels of these K-CRGs were down-regulated after CuCl_2_ treatment, while these trends were significantly weakened after treatment with GSH combined with CuCl_2_ (Figure 14A–14D). These results indicated that the expression levels of the K-CRGs were down-regulated when cuproptosis occurs. The downregulation of these genes in patients with primary myocardiopathy has been found in previous bioinformatics analysis, which indicated that cuproptosis may be involved in the pathogenesis of primary cardiomyopathy.

### Effects of K-CRGs on cuproptosis in AC16 cells

**Figure 15:**
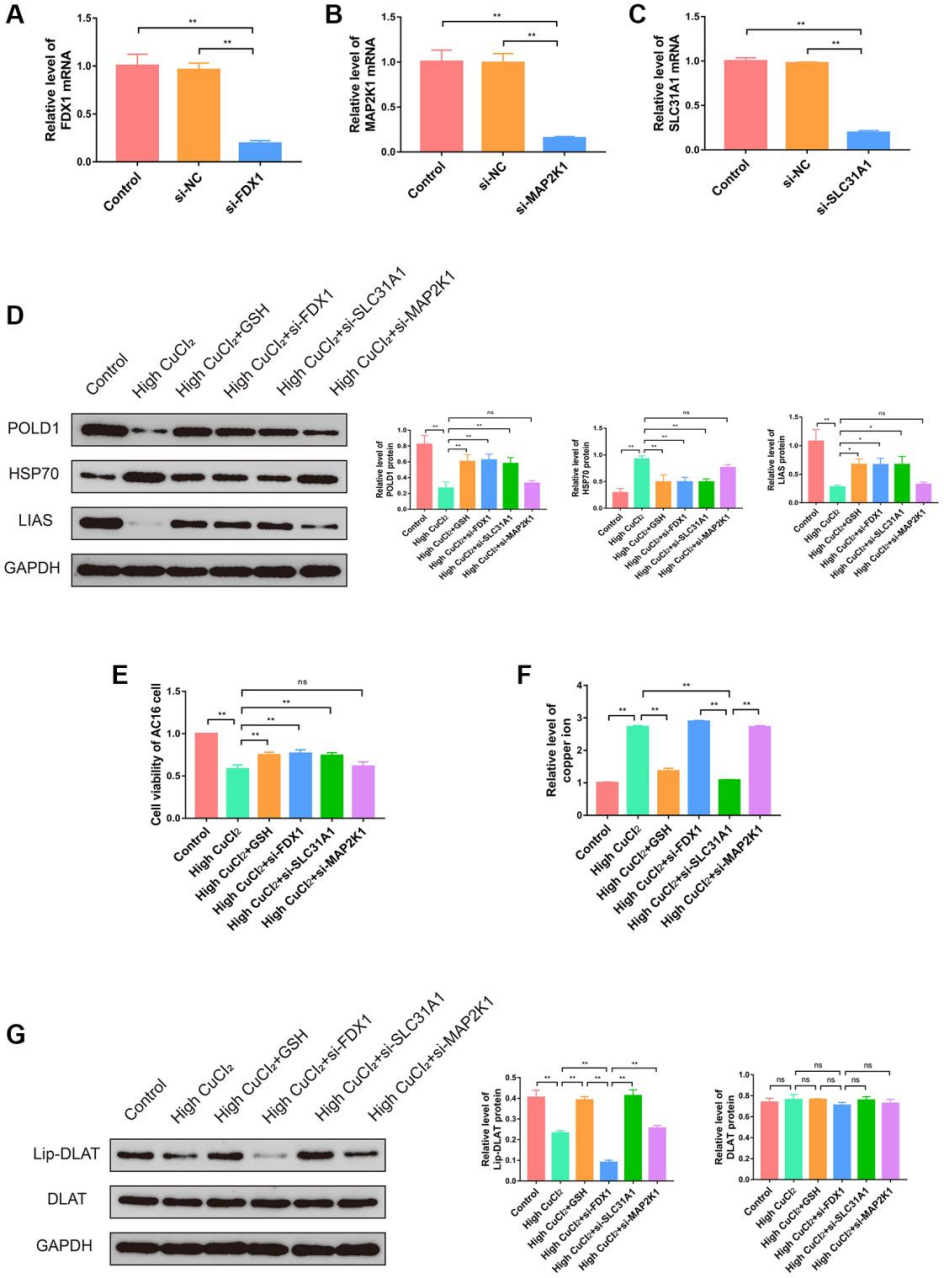
**Effects of three K-CRGs on cuproptosis in AC16 cells.** (**A**–**C**) The effect of siRNA knockdown on three K-CRGs. (**D**) The protein expression levels of POLD1, HSP70 and LIAS in AC16 cells after three K-CRGs were knocked down (*n* = 3). (**E**) The effects of three K-CRGs knockdown on AC16 cells viability. (**F**) The effects of three K-CRGs knockdown on copper ion levels in AC16 cells. (**G**) The protein expression levels of lipoylated DLAT and total DLAT in AC16 cells after three K-CRGs were knocked down (*n* = 3). (^*^P < 0.05, ^**^P < 0.01).

To verify the role of three K-CRGs in cuproptosis of AC16 cells, we interfered with the expression levels of three K-CRGs through siRNA and then observed the changes in cuproptosis. Firstly, we demonstrated that siRNA could significantly down-regulate the expression levels of three K-CRGs in AC16 cells (Figure 15A–15C). After that, the changes in cuproptosis marker proteins were tested in AC16 cells after knockdown of three K-CRGs to embody the degree of cuproptosis. The results indicated that the cuproptosis degree was declined after knockdown of FDX1 and SLC31A1 (specifically manifested as the protein expression levels of LIAS and POLD1 were up-regulated, and HSP70 were down-regulated), while the cuproptosis degree was not significantly changed after knockdown of MAP2K1 (Figure 15D). These results suggested that expression levels of FDX1 and SLC31A1 might impact the cuproptosis degree, the expression level of MAP2K1 only changed with the change of cuproptosis degree, but can’t impact the cuproptosis degree. Then, we tested the cell viability after knockdown of three K-CRGs. The results indicated that after knockdown of FDX1 and SLC31A1, the viability of AC16 cells was significantly increased, while the viability of AC16 cells did not change after knockdown of MAP2K1 (Figure 15E). These results further demonstrated FDX1 and SLC31A1 could affect the severity of cuproptosis in AC16 cells and thereby impact the cell viability. Subsequently, we further explored the mechanisms by which the FDX1 gene and SLC31A1 gene regulate cuproptosis in cardiomyocytes. According to previous literature reports, the core pathological mechanism of cuproptosis is as follows: the copper ion binds to lipoylated DLAT protein, inducing the aggregation of a large amount of copper and lipoylated DLAT complexes in the cell, eventually resulting in proteotoxic stress and cell death [11]. It should be noted that the lipoylation of DLAT is a necessary condition for binding copper ions, and DLAT that is not modified by lipoylation cannot bind to copper ions. Therefore, intracellular copper ion level and lipoylation level of DLAT are the key factors affecting cuproptosis. Our next experiments attempted to explore whether FDX1 gene and SLC31A1 gene affect cuproptosis in cardiomyocytes through the regulation of copper ion level and DLAT lipoylation level. The results showed that, compared with CuCI_2_ treatment group, knockdown of SLC31A1 gene could significantly down-regulate copper ion level, knockdown of FDX1 gene could significantly reduce the level of lipoylated DLAT and had no significant effect on total DLAT level, knockdown of MAP2K1 gene had no significant effect on copper ion level and lipoylated DLAT level (Figure 15F, 15G). These results suggested that SLC31A1 gene affected the degree of cuproptosis in cardiomyocyte mainly by regulating the level of intracellular copper ion, and FDX1 gene affected the degree of cuproptosis in cardiomyocyte mainly by regulating the level of lipoylated DLAT. In the future, the SLC31A1 gene and FDX1 gene are expected to be potential therapeutic targets for primary cardiomyopathy.

## DISCUSSION

The pathogenesis of a variety of primary cardiomyopathy, including DCM, HCM and ARVC, has not been fully elucidated at present, resulting in the research on early diagnosis and targeted therapy has not achieved breakthrough progress. Programmed cell death plays an important role in the course of many kinds of primary cardiomyopathy and is expected to clarify the exact pathogenesis of various types of primary cardiomyopathy and the potential relationship between them [27]. The copper is one of the significant cofactors of transporters and enzymes. The copper has a role in maintaining cell redox balance, and recent research has found that copper could induce a unique mode of programmed cell death, known as cuproptosis [11]. Previous investigation had reported that dysmetabolism of copper could cause heart failure and cardiomyopathy, but the exact mechanism remained unclear [28, 29]. Now, cuproptosis offers the probability to reveal the potential mechanism. Although cuproptosis is a mode of cell death that has only recently been reported, there are many studies that have revealed the correlation between cuproptosis and multiple diseases [30, 31]. As far as we know, our research is the first to report that cuproptosis is the potential common pathogenesis of three kinds of primary cardiomyopathy.

In our study, multiple bioinformatics analysis methods were employed to perform a comprehensive analysis of six GEO datasets of primary cardiomyopathy, including 749 ventricular tissue samples. We first identified four shared differential CRGs that were significantly differentially expressed in all three kinds of primary cardiomyopathy. These four CRGs contained two core genes (FDX1 and SLC31A1) that regulate cuproptosis, suggesting that cuproptosis may be the common pathogenesis of the three kinds of primary cardiomyopathy. In subsequent analysis, we identified three K-CRGs with the best diagnostic performance. The machine learning models constructed based on the three K-CRGs not only exhibited excellent diagnostic performance in the three kinds of primary cardiomyopathy, but also exhibited excellent performance in the identification of molecular subtype. Previous research had found that patients with certain diseases could be divided into different molecular subtypes clusters based on differences in gene expression profiles. The different molecular subtypes clusters have different clinical features, therapy sensitivity and prognosis [32–34]. In our research, we discovered that K-CRGs were able to identify different clusters of molecular subtypes in primary cardiomyopathy. The different clusters of molecular subtypes in each cardiomyopathy differed in the biological functions associated with cell death and immune inflammation. This suggested that K-CRGs held promise for identifying subpopulations with specific cardiomyocyte death and immunoinflammatory signatures in patients with primary cardiomyopathy. Cardiomyocyte death and immunoinflammation are the pathogenesis of primary cardiomyopathy, therefore, patients with different molecular subtypes may differ in disease severity and may show different sensitivities to anti-inflammatory and anti-cell death therapies. It is reasonable to expect that the value of these K-CRGs in the identification of molecular subtypes will contribute to the development of risk stratification and individualized treatment strategies for patients with primary cardiomyopathy in the future. In addition, we built the ceRNA network and targeted drug network of the K-CRGs on the basis of the online databases, which will contribute to the future research of therapeutic targets and drugs. Our study not only found the latent relevance between primary cardiomyopathy and cuproptosis, but also offered valuable evidence for the use of CRGs in the diagnosis and molecular subtype identification of primary cardiomyopathy. Further study on cuproptosis and primary cardiomyopathy is expected to clarify the pathogenesis of primary cardiomyopathy, find new diagnostic markers and individualized therapy methods. However, what needs to be pointed out is that the differential genes selection standard employed in our research was relatively lax. It is well known that when conducting bioinformatics analysis, differential expression analysis of tumor diseases often generates many differential genes, but the amounts of differential genes acquired in analyses of non-tumor diseases is usually very small. In this case, it is inapposite to employ strict selection standard, such as |log2 fold change| > 1. Some research of bioinformatics analysis defined the *P*-value less than 0.05 as the selection standard for differential genes [35, 36]. Therefore, this standard was also adopted in our study. It should be acknowledged that such selection standard can lead to higher false positive rates.

The SLC31A1 gene encodes a cupric transporter protein, which performs cupric transmembrane transport in trimer form and plays an important role in regulating cupric homeostasis [37]. In addition, SLC31A1 also mediates cell uptake of platinum chemotherapy drugs, so previous studies on SLC31A1 mainly focused on tumor drug resistance [38]. Recently, researchers have noticed the role of SLC31A1 in cardiomyopathy. One study reported that SLC31A1-related signaling pathways may be involved in the pathogenesis of diabetic cardiomyopathy [39]. MAP2K1 is a member of the dual specificity protein kinase family and is an important part of the intracellular and extracellular MAP kinase signaling pathway, which is involved in many biological processes such as cell proliferation and differentiation [40]. Studies have found that the function of MAP2K1 depends on the activation of copper [41], and the MAP2K1-mediated signaling pathway could lead to myocardial myofibrillar disarray and myocardial hypertrophy [42]. Meanwhile, MAP2K1 inhibitors could rescue myocardial hypertrophy and fibrosis and improve cardiac function [43]. These findings suggest that MAP2K1 is potentially associated with cuproptosis and cardiomyopathy. The FDX1 gene encodes a small molecular iron-sulfur clusters protein, which plays an important role in regulating steroid hormone synthesis, mitochondrial cytochrome P450 enzyme electron transfer, and lipoylation [44, 45]. The recent study had found that FDX1-mediated protein lipoylation is the key link in the occurrence of cuproptosis [11]. Previous studies have never reported the role of FDX1 in cardiomyopathy or heart failure. Our study is the first to find that FDX1 is significantly down-regulated in three kinds of primary cardiomyopathy, suggesting that FDX1 may be involved in the pathogenesis of primary cardiomyopathy through cuproptosis mechanism. Next, we performed *in vitro* experiments to further investigate the role of these three genes in cuproptosis of cardiomyocytes. Since the primary cardiomyopathy is characterized by pathological changes in the heart structure caused by no clear reason, a suitable animal model cannot be established. Previous studies have chemically induced cardiac structural changes similar to those in DCM or HCM and found that myocardial programmed cell death plays an important role, such as apoptosis, pyroptosis and ferroptosis [10]. Therefore, our *in vitro* experiment focused on verifying whether cardiomyocytes could lead to death through the cuproptosis mechanism, whether the changes in the expression levels of three K-CRGs when cuproptosis occurred were consistent with the changes in GEO datasets, and whether knockdown of these genes could inhibit the degree of cuproptosis in cardiomyocytes. The results showed that, under certain conditions, cardiomyocytes could be induced to cuproptosis. The mRNA and protein levels of FDX1, MAP2K1 and SLC31A1 were significantly down-regulated during cuproptosis in cardiomyocytes, which was in line with the expression trends of the three kinds of primary cardiomyopathy groups in the GEO datasets. These results indicated that cardiomyocytes cuproptosis may be the common pathogenesis of three kinds of primary cardiomyopathy. Finally, by knocking down these three K-CRGs, we further found that FDX1 and SLC31A1 could regulate the degree of cuproptosis in cardiomyocytes and affect cell viability. These results indicated that these two genes have the potential to be new therapeutic targets for primary cardiomyopathy in the future, which needs to be confirmed in further studies.

Our research contained a large sample size and is the first to report the potential relevance between cuproptosis and primary cardiomyopathy. Thus, our research offered compelling basis for the discovery of novel markers for primary cardiomyopathy diagnosis and molecular subtypes identification. At the same time, our research also broke new ground for future studies on the pathogenesis and targeted therapy of primary cardiomyopathy. However, we should admit that our research has certain limitations: First, the detection of indicators related to cuproptosis *in vitro* experiments was not comprehensive enough. In our research, the expression levels of three cuproptosis signature proteins were employed to embody the degree of cuproptosis, but did not detect other indicators that could embody the degree of cuproptosis. Second, since the primary cardiomyopathy is characterized by pathological changes in the heart structure caused by no clear reason, a suitable animal model cannot be established. In this study, only *in vitro* experiments were conducted to verify the results of bioinformatics analysis, and further *in vivo* experiments were lacking for more adequate verification. We believe we will refine the limitations in further research.

In conclusion, by bioinformatics analysis and *in vitro* experimental validation, our research found that cuproptosis possibly be the potential common pathogenesis of three kinds of primary cardiomyopathy, and FDX1, MAP2K1 and SLC31A1 possibly be prospective markers for the diagnosis and molecular subtype identification of primary cardiomyopathy. Our research has broken new ground for the domain of primary cardiomyopathy, further investigation on the foundation of our research is expected to make inspiring progress in the clarification of pathogenesis and development of novel diagnosis and therapy approaches of primary cardiomyopathy in the future.

## Supplementary Materials

Supplementary Figures

Supplementary Tables 1-3

Supplementary Table 4
