# Supplementary material for: Integrated bioinformatics and experiment revealed that cuproptosis is the potential common pathogenesis of three kinds of primary cardiomyopathy
Source: Aging (Albany NY). 2023 Dec 11;15(23):14210–41. doi: 10.18632/aging.205298 (PMC10756114; doi:10.18632/aging.205298)
Supplement: Supplementary Figures [file aging-15-205298-s001.pdf]

SUPPLEMENTARY FIGURES

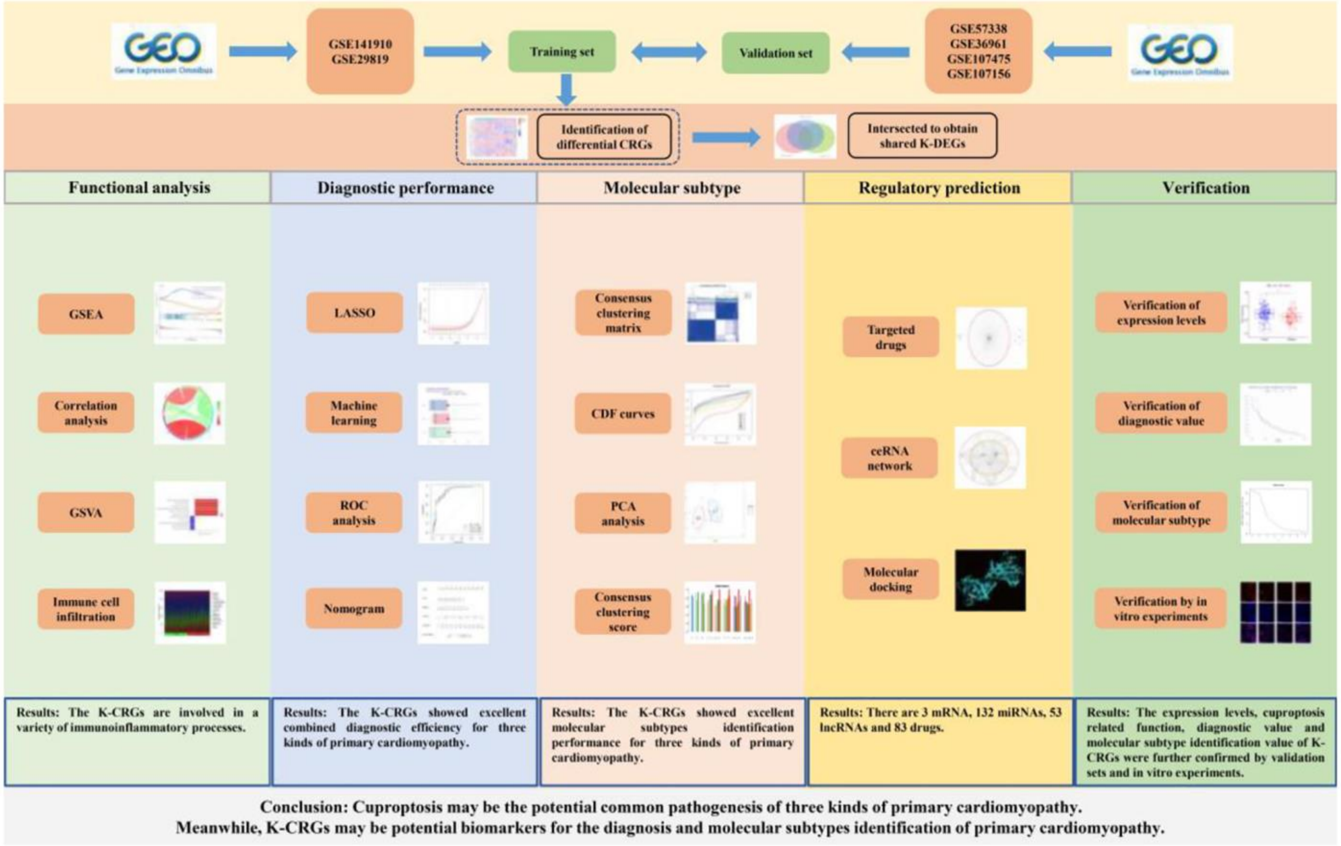

Supplementary Figure 1. The flowchart of this study.

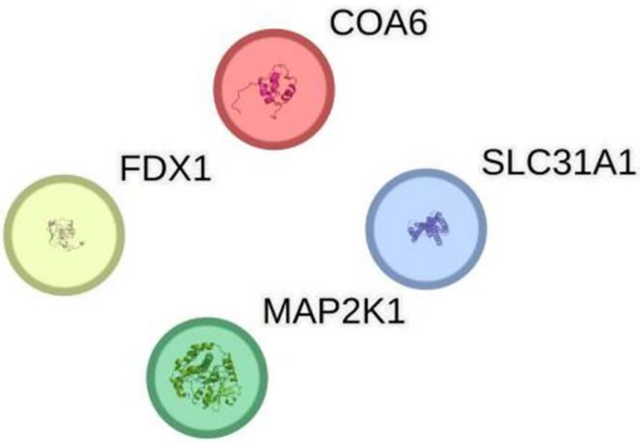

Supplementary Figure 2. The PPI network of four CRGs shared by the three kinds of primary cardiomyopathy.

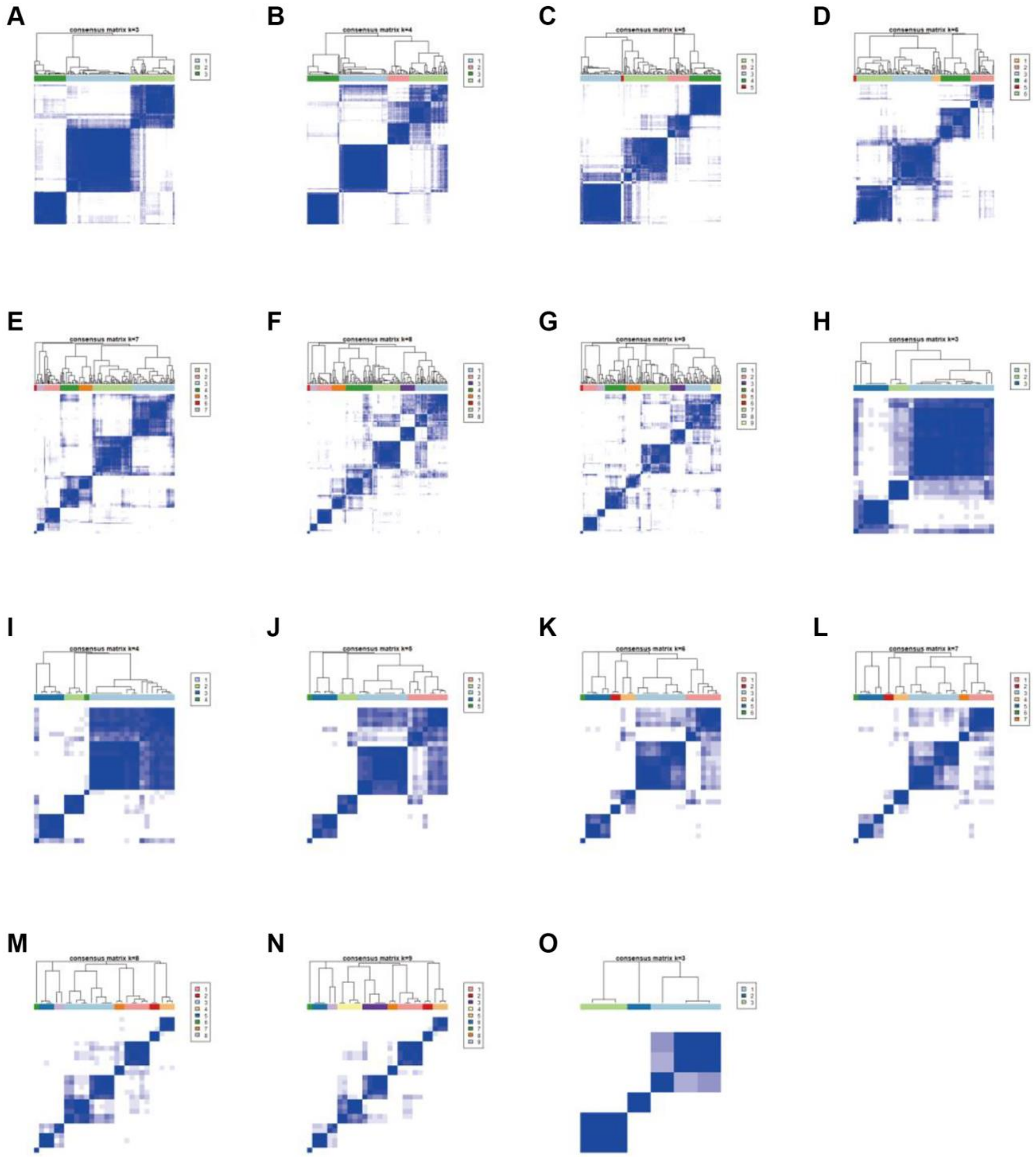

Supplementary Figure 3. The consensus clustering matrix of three kinds of primary cardiomyopathy when k = 3-9. (**A**–**G**) k = 3 − 9 of DCM. (**H**–**N**) k = 3 − 9 of HCM. (**O**) k = 3 of ARVC.

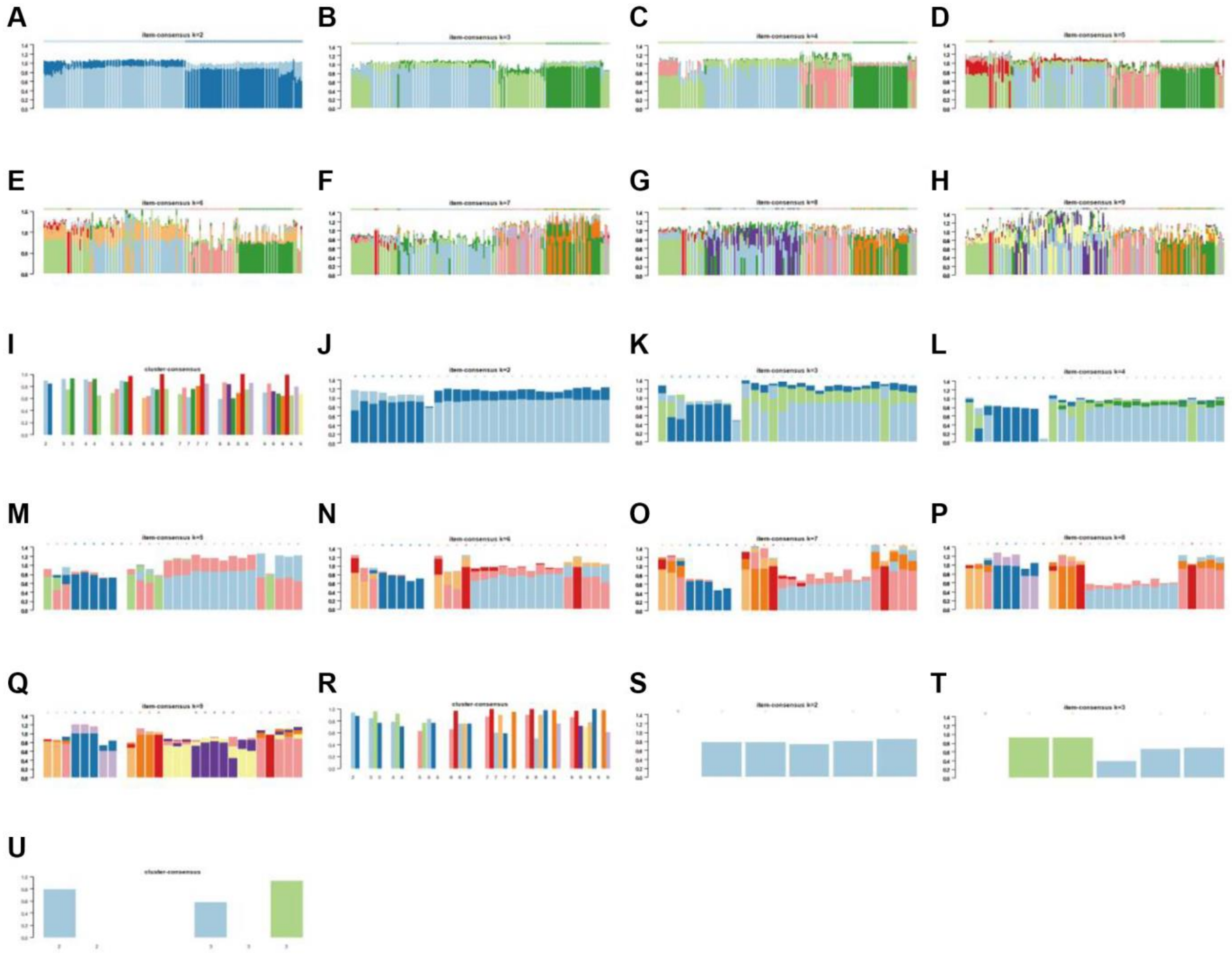

Supplementary Figure 4. The item and score of consensus clustering of three kinds of primary cardiomyopathy when k = 2 − 9. (**A**–**H**) The item of consensus clustering of DCM when k = 2 − 9. (**I**) The score of consensus clustering of DCM when k = 2 − 9. (**J**–**Q**) The item of consensus clustering of HCM when k = 2 − 9. (**R**) The score of consensus clustering of HCM when k = 2 − 9. (**S**, **T**) The item of consensus clustering of ARVC when k = 2 − 3. (**U**) The score of consensus clustering of ARVC when k = 2 − 3.

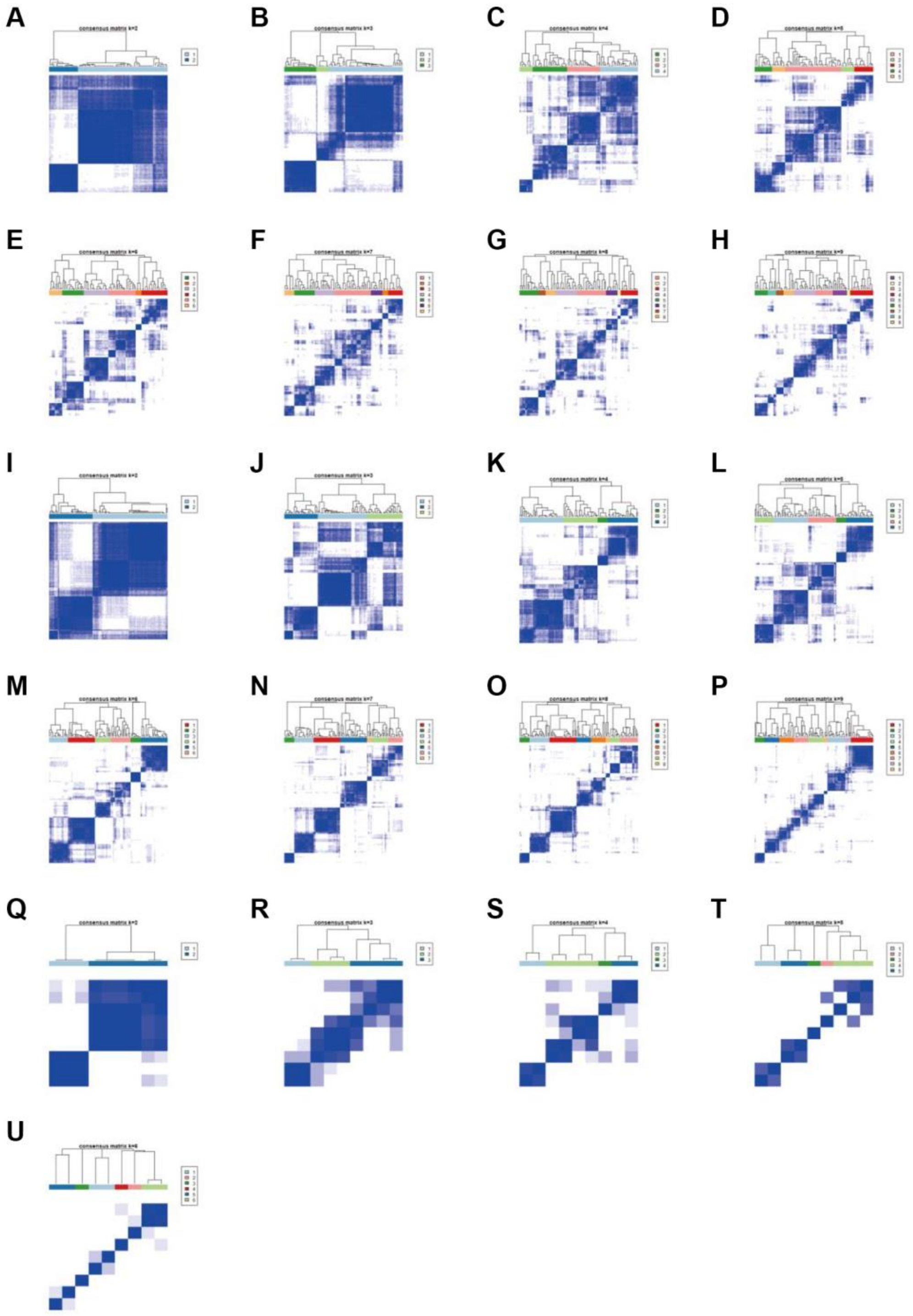

Supplementary Figure 5. The consensus clustering matrix of three kinds of primary cardiomyopathy when k = 2 − 9 in the validation set. (**A**–**H**) k = 2 − 9 of DCM. (**I**–**P**) k = 2 − 9 of HCM. (**Q**–**U**) k = 2 − 6 of ARVC.

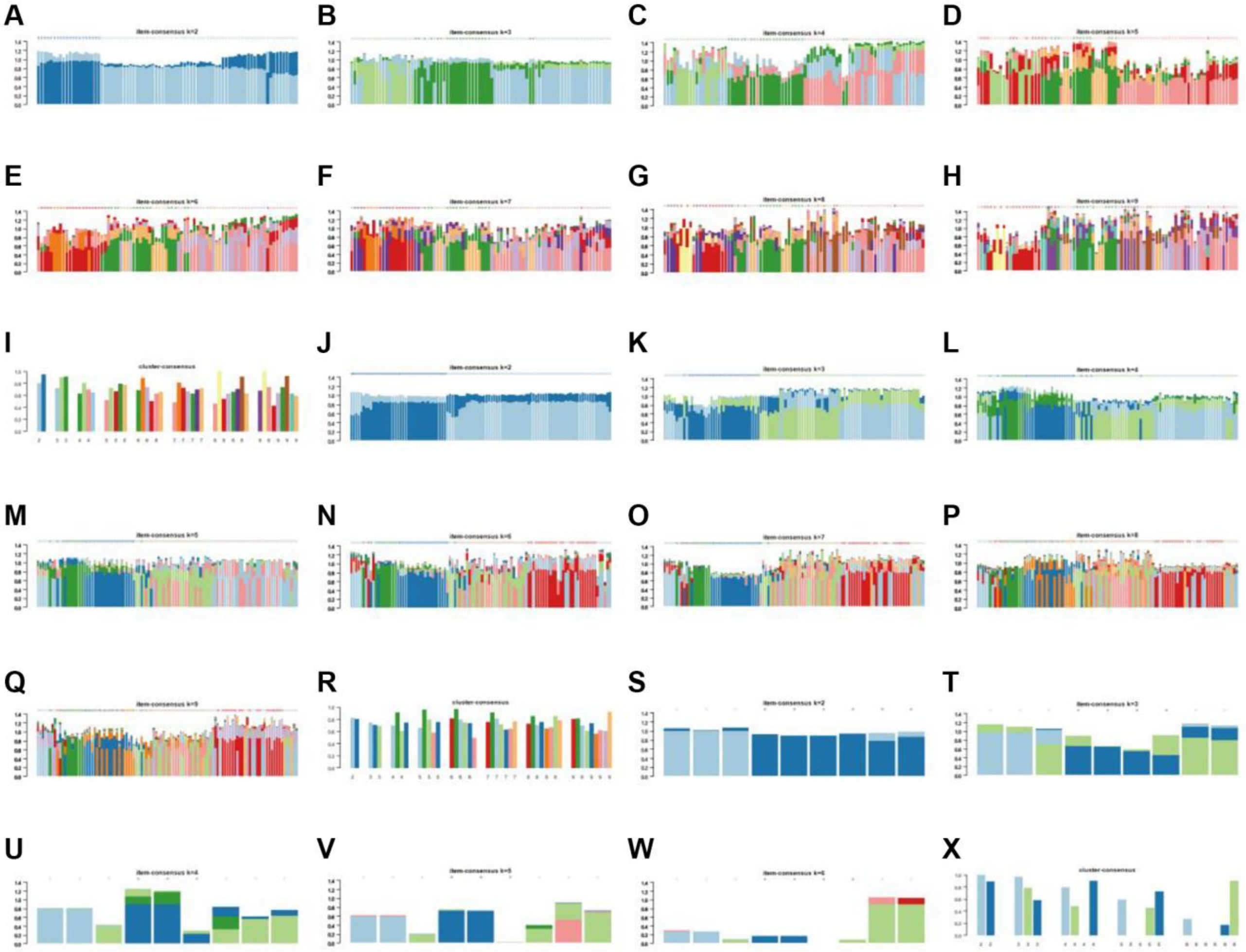

Supplementary Figure 6. The item and score of consensus clustering of three kinds of primary cardiomyopathy when k = 2 − 9 in the validation set. (**A**–**H**) The item of consensus clustering of DCM when k = 2 − 9. (**I**) The score of consensus clustering of DCM when k = 2 − 9. (**J**–**Q**) The item of consensus clustering of HCM when k = 2 − 9. (**R**) The score of consensus clustering of HCM when k = 2 − 9. (**S**–**W**) The item of consensus clustering of ARVC when k = 2 − 6. (**X**) The score of consensus clustering of ARVC when k = 2 − 6.
