# Supplementary material for: Integrated bioinformatics and experiment revealed that cuproptosis is the potential common pathogenesis of three kinds of primary cardiomyopathy
Source: Aging (Albany NY). 2023 Dec 11;15(23):14210–41. doi: 10.18632/aging.205298 (PMC10756114; doi:10.18632/aging.205298)
Supplement: Supplementary Tables 1-3 [file aging-15-205298-s002.pdf]

SUPPLEMENTARY TABLES

Supplementary Table 1. Summary of six GEO datasets.

Name	Disease type	Disease samples	Control samples	Group
GSE141910	DCM	166	166	Training set
GSE141910	HCM	28	166	Training set
GSE29819	ARVC	6	6	Training set
GSE57338	DCM	82	136	Validation set
GSE36961	HCM	106	39	Validation set
GSE107475	ARVC	9	0	Validation set
GSE107156	ARVC	0	5	Validation set

Abbreviations: DCM: dilated cardiomyopathy; HCM: hypertrophic cardiomyopathy; ARVC: arrhythmogenic right ventricular cardiomyopathy.

Supplementary Table 2. Summary of CRGs symbol.

	CRGs ID	
AOC3	LOXL2	SCO2
ATOX1	MAP2K1	SLC25A3
ATP7A	MAP2K2	SLC31A1
ATP7B	MT1A	SLC31A2
CCL8	MT1B	SOD1
CCS	MT1E	TYR
CD274	MT1F	UBE2D1
CDKN2A	MT1G	UBE2D2
COA6	MT1H	UBE2D3
COX11	MT1M	UBE2D4
COX17	MT1X	ULK1
CP	MT2A	ULK2
DBH	MT-CO1	VEGFA
DLAT	MT-CO2	NFE2L2
DLD	MTF1	NLRP3
FDX1	PDE3B	LIPT2
GLS	PDHA1	DLST
HIST1H3A	PDHB	DBT
LIAS	PDK1	GCSH
LIPT1	SCO1	

Supplementary Table 3. Primer sequences.

Name	Species	Forward primer	Reverse primer
FDX1	Human	TTCAACCTGTCACCTCATCTTTG	TGCCAGATCGAGCATGTCATT
MAP2K1	Human	CAATGGCGGTGTGGTGTTC	GATTGCGGGTTTGATCTCCAG
SLC31A1	Human	GGGGATGAGCTATATGGACTCC	TCACCAAACCGGAAAACAGTAG
GAPDH	Human	GGACCTGACCTGCCGTCTAG	GTAGCCCAGGATGCCCTTGA
