# Supplementary material for: Integrated bioinformatics and experiment revealed that cuproptosis is the potential common pathogenesis of three kinds of primary cardiomyopathy
Source: Aging (Albany NY). 2023 Dec 11;15(23):14210–41. doi: 10.18632/aging.205298 (PMC10756114; doi:10.18632/aging.205298)
Supplement: Supplementary Table 4 [file aging-15-205298-s003.docx]

**Supplementary Table 4. Interactions of ceRNA.**

| **Name of miRNA** | **Name of interacting**  **mRNA or lncRNA** | **Interaction type** |
| --- | --- | --- |
| hsa-miR-4273  hsa-miR-551b-5p  hsa-miR-758-3p  hsa-miR-548s  hsa-miR-34c-5p  hsa-miR-29b-3p  hsa-miR-616-3p  hsa-miR-144-3p  hsa-miR-2110  hsa-miR-944  hsa-let-7i-5p  hsa-miR-590-5p  hsa-miR-149-3p  hsa-miR-101-5p  hsa-miR-148b-3p  hsa-miR-340-5p  hsa-miR-29c-3p  hsa-miR-105-5p  hsa-miR-576-5p  hsa-miR-449b-5p  hsa-miR-641  hsa-miR-330-3p  hsa-miR-127-5p  hsa-miR-522-3p  hsa-miR-495-3p  hsa-miR-501-5p  hsa-miR-936  hsa-miR-761  hsa-miR-4301  hsa-miR-646  hsa-miR-1323  hsa-miR-4322  hsa-let-7a-5p  hsa-miR-148a-3p  hsa-miR-3163  hsa-miR-548g-3p  hsa-miR-583  hsa-miR-760  hsa-miR-890  hsa-miR-4279  hsa-miR-3154  hsa-miR-188-3p  hsa-miR-576-3p  hsa-miR-1179  hsa-miR-3166  hsa-miR-545-3p  hsa-miR-4282  hsa-miR-509-3-5p  hsa-miR-1207-3p  hsa-miR-29a-3p  hsa-let-7b-5p  hsa-miR-3144-3p  hsa-miR-506-3p  hsa-miR-633  hsa-miR-4291  hsa-miR-590-3p  hsa-miR-576-5p  hsa-miR-98-5p  hsa-miR-524-5p  hsa-miR-212-3p  hsa-miR-3123  hsa-miR-145-3p  hsa-miR-922  hsa-miR-4310  hsa-miR-197-3p  hsa-miR-497-5p  hsa-miR-3120-3p  hsa-miR-30b-3p  hsa-miR-30b-3p  hsa-miR-4296  hsa-miR-369-3p  hsa-miR-9-3p  hsa-miR-195-5p  hsa-miR-2054  hsa-miR-613  hsa-let-7g-5p  hsa-miR-508-3p  hsa-miR-222-5p  hsa-miR-196a-5p  hsa-miR-181c-5p  hsa-miR-588  hsa-miR-760  hsa-miR-4311  hsa-miR-221-5p  hsa-let-7f-5p  hsa-miR-518d-5p  hsa-miR-3147  hsa-miR-1256  hsa-miR-335-3p  hsa-miR-1284  hsa-miR-3171  hsa-miR-1-3p  hsa-miR-335-3p  hsa-let-7e-5p  hsa-miR-646  hsa-miR-606  hsa-miR-138-2-3p  hsa-miR-181b-5p  hsa-miR-3130-5p  hsa-miR-132-3p  hsa-miR-629-5p  hsa-miR-96-5p  hsa-miR-34b-3p  hsa-miR-4265  hsa-miR-505-3p  hsa-miR-214-3p  hsa-miR-4259  hsa-miR-3148  hsa-let-7d-5p  hsa-miR-4314  hsa-miR-22-5p  hsa-miR-16-5p  hsa-miR-4323  hsa-miR-3149  hsa-miR-15b-5p  hsa-miR-130a-5p  hsa-miR-548a-3p  hsa-miR-3187-3p  hsa-miR-22-5p  hsa-miR-373-5p  hsa-miR-181a-5p  hsa-miR-449a  hsa-miR-34a-5p  hsa-miR-21-5p  hsa-miR-485-3p  hsa-miR-3130-5p  hsa-miR-424-5p  hsa-miR-15a-5p  hsa-miR-125a-3p  hsa-miR-1271-5p  hsa-miR-4284  hsa-miR-509-5p  hsa-miR-509-3p  hsa-miR-196b-5p  hsa-miR-497-3p  hsa-miR-622  hsa-miR-570-3p  hsa-miR-197-3p  hsa-miR-206  hsa-miR-503-5p | FDX1  SLC31A1  MAP2K1  SLC31A1  MAP2K1  SLC31A1  SLC31A1  MAP2K1  MAP2K1  FDX1  SLC31A1  FDX1  FDX1  FDX1  MAP2K1  FDX1  SLC31A1  SLC31A1  FDX1  MAP2K1  FDX1  MAP2K1  FDX1  MAP2K1  FDX1  MAP2K1  MAP2K1  SLC31A1  SLC31A1  SLC31A1  MAP2K1  SLC31A1  SLC31A1  MAP2K1  FDX1  SLC31A1  SLC31A1  MAP2K1  MAP2K1  SLC31A1  SLC31A1  MAP2K1  SLC31A1  FDX1  SLC31A1  MAP2K1  FDX1  SLC31A1  SLC31A1  SLC31A1  SLC31A1  SLC31A1  SLC31A1  MAP2K1  SLC31A1  FDX1  SLC31A1  SLC31A1  SLC31A1  SLC31A1  FDX1  SLC31A1  SLC31A1  FDX1  SLC31A1  MAP2K1  SLC31A1  FDX1  SLC31A1  SLC31A1  FDX1  FDX1  MAP2K1  FDX1  SLC31A1  SLC31A1  SLC31A1  FDX1  SLC31A1  MAP2K1  SLC31A1  SLC31A1  SLC31A1  MAP2K1  SLC31A1  SLC31A1  SLC31A1  FDX1  SLC31A1  MAP2K1  SLC31A1  SLC31A1  MAP2K1  SLC31A1  MAP2K1  FDX1  SLC31A1  MAP2K1  SLC31A1  SLC31A1  SLC31A1  MAP2K1  FDX1  SLC31A1  SLC31A1  SLC31A1  SLC31A1  SLC31A1  SLC31A1  SLC31A1  MAP2K1  MAP2K1  MAP2K1  SLC31A1  MAP2K1  FDX1  SLC31A1  SLC31A1  SLC31A1  SLC31A1  MAP2K1  MAP2K1  MAP2K1  FDX1  MAP2K1  FDX1  MAP2K1  MAP2K1  SLC31A1  MAP2K1  FDX1  SLC31A1  SLC31A1  SLC31A1  SLC31A1  FDX1  FDX1  MAP2K1  SLC31A1  MAP2K1 | miRNA-mRNA  miRNA-mRNA  miRNA-mRNA  miRNA-mRNA  miRNA-mRNA  miRNA-mRNA  miRNA-mRNA  miRNA-mRNA  miRNA-mRNA  miRNA-mRNA  miRNA-mRNA  miRNA-mRNA  miRNA-mRNA  miRNA-mRNA  miRNA-mRNA  miRNA-mRNA  miRNA-mRNA  miRNA-mRNA  miRNA-mRNA  miRNA-mRNA  miRNA-mRNA  miRNA-mRNA  miRNA-mRNA  miRNA-mRNA  miRNA-mRNA  miRNA-mRNA  miRNA-mRNA  miRNA-mRNA  miRNA-mRNA  miRNA-mRNA  miRNA-mRNA  miRNA-mRNA  miRNA-mRNA  miRNA-mRNA  miRNA-mRNA  miRNA-mRNA  miRNA-mRNA  miRNA-mRNA  miRNA-mRNA  miRNA-mRNA  miRNA-mRNA  miRNA-mRNA  miRNA-mRNA  miRNA-mRNA  miRNA-mRNA  miRNA-mRNA  miRNA-mRNA  miRNA-mRNA  miRNA-mRNA  miRNA-mRNA  miRNA-mRNA  miRNA-mRNA  miRNA-mRNA  miRNA-mRNA  miRNA-mRNA  miRNA-mRNA  miRNA-mRNA  miRNA-mRNA  miRNA-mRNA  miRNA-mRNA  miRNA-mRNA  miRNA-mRNA  miRNA-mRNA  miRNA-mRNA  miRNA-mRNA  miRNA-mRNA  miRNA-mRNA  miRNA-mRNA  miRNA-mRNA  miRNA-mRNA  miRNA-mRNA  miRNA-mRNA  miRNA-mRNA  miRNA-mRNA  miRNA-mRNA  miRNA-mRNA  miRNA-mRNA  miRNA-mRNA  miRNA-mRNA  miRNA-mRNA  miRNA-mRNA  miRNA-mRNA  miRNA-mRNA  miRNA-mRNA  miRNA-mRNA  miRNA-mRNA  miRNA-mRNA  miRNA-mRNA  miRNA-mRNA  miRNA-mRNA  miRNA-mRNA  miRNA-mRNA  miRNA-mRNA  miRNA-mRNA  miRNA-mRNA  miRNA-mRNA  miRNA-mRNA  miRNA-mRNA  miRNA-mRNA  miRNA-mRNA  miRNA-mRNA  miRNA-mRNA  miRNA-mRNA  miRNA-mRNA  miRNA-mRNA  miRNA-mRNA  miRNA-mRNA  miRNA-mRNA  miRNA-mRNA  miRNA-mRNA  miRNA-mRNA  miRNA-mRNA  miRNA-mRNA  miRNA-mRNA  miRNA-mRNA  miRNA-mRNA  miRNA-mRNA  miRNA-mRNA  miRNA-mRNA  miRNA-mRNA  miRNA-mRNA  miRNA-mRNA  miRNA-mRNA  miRNA-mRNA  miRNA-mRNA  miRNA-mRNA  miRNA-mRNA  miRNA-mRNA  miRNA-mRNA  miRNA-mRNA  miRNA-mRNA  miRNA-mRNA  miRNA-mRNA  miRNA-mRNA  miRNA-mRNA  miRNA-mRNA  miRNA-mRNA  miRNA-mRNA  miRNA-mRNA  miRNA-mRNA |
| hsa-miR-449a  hsa-miR-449a  hsa-miR-449a  hsa-miR-449a  hsa-miR-449a  hsa-miR-449a  hsa-miR-449a  hsa-miR-449a  hsa-miR-449a  hsa-miR-449a  hsa-miR-449a  hsa-miR-449a  hsa-miR-148b-3p  hsa-miR-148b-3p  hsa-miR-148b-3p  hsa-miR-148b-3p  hsa-miR-148b-3p  hsa-miR-148b-3p  hsa-miR-148b-3p  hsa-miR-148b-3p  hsa-miR-148b-3p  hsa-miR-148b-3p  hsa-miR-148b-3p  hsa-miR-148a-3p  hsa-miR-148a-3p  hsa-miR-148a-3p  hsa-miR-148a-3p  hsa-miR-148a-3p  hsa-miR-148a-3p  hsa-miR-148a-3p  hsa-miR-148a-3p  hsa-miR-206  hsa-miR-206  hsa-miR-206  hsa-miR-206  hsa-miR-206  hsa-miR-206  hsa-miR-206  hsa-miR-206  hsa-miR-206  hsa-miR-206  hsa-miR-206  hsa-miR-34c-5p  hsa-miR-34c-5p  hsa-miR-34c-5p  hsa-miR-508-3p  hsa-miR-508-3p  hsa-miR-508-3p  hsa-miR-508-3p  hsa-miR-508-3p  hsa-miR-508-3p  hsa-miR-508-3p  hsa-miR-761  hsa-miR-761  hsa-miR-761  hsa-miR-761  hsa-miR-761  hsa-miR-761  hsa-miR-761  hsa-miR-761  hsa-miR-761  hsa-miR-761  hsa-miR-761  hsa-miR-761  hsa-miR-761  hsa-miR-761  hsa-miR-761  hsa-miR-212-3p  hsa-miR-212-3p  hsa-miR-212-3p  hsa-miR-212-3p  hsa-miR-212-3p  hsa-miR-613  hsa-miR-613  hsa-miR-613  hsa-miR-613  hsa-miR-613  hsa-miR-613  hsa-miR-613  hsa-miR-613  hsa-miR-613  hsa-miR-613  hsa-miR-590-5p  hsa-miR-590-5p | ERVH48-1  KCNQ1OT1  LINC00278  EAF1-AS1  MUC19  CNOT10-AS1  JPX  UBE2Q1-AS1  XIST  NAV2-AS5  KCNA3  SHANK3  OIP5-AS1  SNHG14  LINC00221  KCNQ1OT1  MIR4313  TTTY10  C21orf88  MCCC1-AS1  ITCH-IT1  DNM3OS  ZNRD1-AS1  OIP5-AS1  SNHG14  LINC00221  KCNQ1OT1  MIR4313  HOTAIRM1  DNM3OS  ITCH-IT1  CASK-AS1  SNORD116-20  KCNQ1OT1  MALAT1  C14orf23  SNHG14  NEAT1  MTUS2-AS2  CCDC39-AS1  MYLK-AS1  ZNRF3-AS1  KCNQ1OT1  LINC00278  MIR4313  TTTY12  KCNQ1OT1  MUC19  ARHGEF26-AS1  ST7-OT4  C21orf128  LINC00517  KCNQ1OT1  ATXN8OS  C1orf132  TTTY4C  TTTY4B  TTTY4  XIST  C21orf90  SHANK3  ZNRD1-AS1  C14orf182  LINC00176  FAM138E  MIR4313  FAM138A  XIST  ZNF503-AS1  KCNQ1OT1  MUC19  NEAT1  SNORD116-20  CASK-AS1  KCNQ1OT1  TIPARP-AS1  C14orf23  MIR22HG  SNHG14  MALAT1  NEAT1  DIAPH3-AS1  FAM66C  TTTY15 | lncRNA-miRNA  lncRNA-miRNA  lncRNA-miRNA  lncRNA-miRNA  lncRNA-miRNA  lncRNA-miRNA  lncRNA-miRNA  lncRNA-miRNA  lncRNA-miRNA  lncRNA-miRNA  lncRNA-miRNA  lncRNA-miRNA  lncRNA-miRNA  lncRNA-miRNA  lncRNA-miRNA  lncRNA-miRNA  lncRNA-miRNA  lncRNA-miRNA  lncRNA-miRNA  lncRNA-miRNA  lncRNA-miRNA  lncRNA-miRNA  lncRNA-miRNA  lncRNA-miRNA  lncRNA-miRNA  lncRNA-miRNA  lncRNA-miRNA  lncRNA-miRNA  lncRNA-miRNA  lncRNA-miRNA  lncRNA-miRNA  lncRNA-miRNA  lncRNA-miRNA  lncRNA-miRNA  lncRNA-miRNA  lncRNA-miRNA  lncRNA-miRNA  lncRNA-miRNA  lncRNA-miRNA  lncRNA-miRNA  lncRNA-miRNA  lncRNA-miRNA  lncRNA-miRNA  lncRNA-miRNA  lncRNA-miRNA  lncRNA-miRNA  lncRNA-miRNA  lncRNA-miRNA  lncRNA-miRNA  lncRNA-miRNA  lncRNA-miRNA  lncRNA-miRNA  lncRNA-miRNA  lncRNA-miRNA  lncRNA-miRNA  lncRNA-miRNA  lncRNA-miRNA  lncRNA-miRNA  lncRNA-miRNA  lncRNA-miRNA  lncRNA-miRNA  lncRNA-miRNA  lncRNA-miRNA  lncRNA-miRNA  lncRNA-miRNA  lncRNA-miRNA  lncRNA-miRNA  lncRNA-miRNA  lncRNA-miRNA  lncRNA-miRNA  lncRNA-miRNA  lncRNA-miRNA  lncRNA-miRNA  lncRNA-miRNA  lncRNA-miRNA  lncRNA-miRNA  lncRNA-miRNA  lncRNA-miRNA  lncRNA-miRNA  lncRNA-miRNA  lncRNA-miRNA  lncRNA-miRNA  lncRNA-miRNA  lncRNA-miRNA |
